# MultiCardioNet: A Multimodal Deep Learning Model for Early Cardiovascular Deterioration Prediction in ICU Patients

**DOI:** 10.3390/bioengineering13090993

**Published:** 2026-08-27

**Authors:** Bassem Jandoubi, Moulay A. Akhloufi

**Affiliations:** Perception, Robotics, and Intelligent Machines (PRIME), Department of Computer Science, Université de Moncton, Moncton, NB E1A3E9, Canada; moulay.akhloufi@umoncton.ca

**Keywords:** multimodal learning, cardiovascular deterioration, intensive care unit, MIMIC-IV, radiology reports, CXR-BERT, vital sign time series, deep learning

## Abstract

Cardiovascular deterioration is a clinically important event in intensive care units, but early prediction remains challenging because risk may be reflected across structured clinical variables, physiological time series, and clinical text. In this work, we present MultiCardioNet, a multimodal deep learning framework for early prediction of a composite ICU deterioration endpoint using MIMIC-IV data. The prediction target was defined as vasopressor initiation and/or early death during the 24–72 h outcome window, making the task broader than mortality prediction alone but also related to treatment escalation and circulatory support. The model combines structured clinical variables, 24-h vital sign time series, and timestamp-filtered radiology reports. Structured information was represented using enriched first-24-h clinical features, while physiological dynamics were modeled using a transformer-based time series encoder and radiology reports were represented using CXR-BERT-specialized embeddings. On the held-out test set, MultiCardioNet achieved an AUROC of 0.9014, AUPRC of 0.8481, and F1-score of 0.7723. These findings suggest that the three modalities provide complementary information for this composite deterioration endpoint in the internal test setting.

## 1. Introduction

Cardiovascular deterioration is a major clinical concern in intensive care units (ICUs), where critically ill patients may rapidly develop hemodynamic instability and require urgent intervention. Such deterioration can be reflected by hypotension, worsening oxygenation, abnormal laboratory values, changes in vital signs, or the need for vasoactive and vasopressor medications. Early identification of patients at risk is important because delayed recognition may reduce the opportunity for timely monitoring, treatment adjustment, and escalation of care. Therefore, the development of predictive tools capable of estimating deterioration risk during the early ICU period is an important objective in critical care research.

The increasing availability of electronic health record (EHR) data has enabled the development of machine learning and deep learning approaches for clinical risk prediction. Large critical care databases such as MIMIC-IV [1] contain structured demographic and clinical variables, laboratory measurements, medication records, physiological time series, and clinical text. These heterogeneous data sources provide complementary views of the patient state. Structured clinical variables summarize important numerical information, while vital sign time series capture temporal physiological dynamics and radiology reports may contain clinical interpretation of cardiopulmonary findings that are not fully represented in structured tables.

Many existing clinical prediction models focus on mortality as the primary outcome [2,3]. Although mortality is an important endpoint, it does not represent all clinically relevant forms of deterioration. A patient may experience severe cardiovascular instability and require vasopressor support without dying during the prediction window. For this reason, mortality prediction alone may miss important cases where the patient deteriorates but survives after intervention. In this study, we focus on a composite cardiovascular deterioration endpoint defined by vasopressor initiation and/or early death. This endpoint is broader than mortality alone because it also captures patients requiring hemodynamic support.

Recent deep learning methods have shown promise in modeling structured EHR data and physiological time series [4,5]. Recurrent neural networks, temporal convolutional networks, and transformer-based architectures [6,7,8] have been used to learn temporal patterns from ICU measurements [9,10,11]. In parallel, clinical language models [12,13] have been used to extract information from clinical notes and radiology reports. However, many models rely on a single modality or combine data sources without carefully separating the information carried by each source [14,15]. This can limit the ability of the model to represent complex ICU deterioration, which may appear differently across laboratory values, physiological trends, and clinical text.

Multimodal learning is a natural approach to integrating these heterogeneous sources of information [16]. Combining structured clinical variables, vital sign dynamics, and radiology-derived text features enables a multimodal model to capture signals related to cardiovascular deterioration that would otherwise be overlooked. However, multimodal clinical prediction also presents significant challenges. Different modalities have different data formats, temporal resolutions, and patterns of missing data. In addition, using clinical text requires careful temporal filtering to avoid outcome leakage. For instance, discharge summaries, are written after hospitalization and may contain information about treatments, complications, and outcomes; thus, using them for early prediction can lead to overly optimistic results.

In this context, we present MultiCardioNet, a multimodal deep learning framework for early prediction of cardiovascular deterioration using MIMIC-IV data. The framework combines structured clinical variables, 24-h vital sign time series, and radiology reports. Structured information is represented using early demographic, vital sign, and laboratory features, including an enriched clinical representation extracted from the first 24 h of ICU data. Temporal physiological information is modeled using a transformer-based encoder. Radiology reports are represented using CXR-BERT-specialized embeddings to capture radiology-specific textual information. Discharge summaries are excluded from the final modeling pipeline in order to reduce the risk of outcome leakage. The gap addressed in this study is the lack of a leakage-controlled multimodal framework that jointly combines enriched first-24-h structured clinical features, 24-h vital sign dynamics, and timestamp-filtered radiology report embeddings for early prediction of a composite ICU deterioration endpoint.

The main contributions of this work are as follows:

- We formulate early cardiovascular deterioration prediction as a composite endpoint based on vasopressor initiation and/or early death, making the task broader than mortality prediction alone.
- We construct a multimodal MIMIC-IV-based prediction pipeline using structured clinical variables, 24-h vital sign time series, and early radiology reports.
- We evaluate unimodal models using structured clinical data, vital sign time series, and radiology reports in order to independently estimate the contribution of each modality. These models include structured logistic regression, random forest, XGBoost, time series sequence models, and radiology text representations based on TF-IDF and CXR-BERT-specialized embeddings.
- We develop MultiCardioNet, a multimodal neural fusion model that combines enriched first-24-h clinical features, a Transformer-based vital-sign time series encoder, and CXR-BERT-specialized radiology report embeddings.
- We exclude discharge summaries from the final modeling pipeline to reduce the risk of outcome leakage and preserve an early prediction setting.

The remainder of this paper is organized as follows: Section 2 reviews related work on ICU deterioration prediction, clinical time series modeling, clinical text representation, and multimodal healthcare AI; Section 3 describes the proposed methodology, including the prediction task, modality-specific representations, and multimodal fusion; Section 4 presents the data sources, cohort construction, preprocessing steps, model configurations, training details, and evaluation metrics; Section 5 reports the experimental results, including the unimodal baselines, proposed multimodal model, and threshold analysis; Section 6 discusses the main findings and future directions; finally, Section 7 concludes the paper.

## 2. Related Work

Machine learning has been widely studied for early warning and clinical risk prediction in hospitalized and intensive care unit (ICU) patients. A systematic review by Linnen et al. [17] compared early warning systems based on statistical modeling with traditional aggregate-weighted scores [18]. Across six studies, models using statistical or machine learning methods showed better discrimination than aggregate-weighted tools, with a mean AUC of 0.80 compared with 0.73, and also generated fewer alerts to identify one true case of deterioration, with a standardized workup-to-detection ratio of 4.9 compared with 7.1. These results suggest that more advanced predictive models could reduce the burden of alerts while improving deterioration detection.

Several studies have focused on ICU deterioration using vital signs and other routinely collected physiological variables. Thiele et al. [19] developed a real-time ICU deterioration model using automatically recorded vital signs. Deterioration was defined as qSOFA ≥ 2 [20], and the best model was an artificial neural network using four vital parameters: systolic blood pressure, respiratory rate, oxygen saturation, and heart rate. The model achieved an AUC of 0.814 with a sensitivity of 0.853 and a specificity of 0.667 for prediction 10 h before deterioration. Random forest, support vector machine, linear discriminant analysis, and logistic regression achieved lower AUC values ranging from 0.762 to 0.781. While these findings support the usefulness of dynamic vital sign data, the models remained limited to a small number of monitoring variables and did not include clinical text or imaging-derived information.

Cardiac arrest prediction has also received substantial attention. Lee et al. [21] proposed a real-time machine learning model for in-hospital cardiac arrest using heart rate variability measures extracted from ECG data in ICU patients. Their light gradient boosting model achieved an AUROC of 0.881 and AUPRC of 0.104 for predicting cardiac arrest within 0.5–24 h. The model outperformed a clinical parameter-based comparison model, which achieved an AUROC of 0.735. This study shows that ECG-derived physiological variability can provide useful early warning information, although its low AUPRC also illustrates the difficulty of predicting rare events such as in-hospital cardiac arrest.

Other studies have combined baseline clinical data with temporal vital sign trajectories. Lee et al. [22] developed a multimodal machine learning approach for in-hospital cardiac arrest prediction using MIMIC-IV and externally validated it using eICU-CRD and a hospital cohort from National Taiwan University Hospital. Their model combined random forest predictions from baseline features with an LSTM model trained on vital sign trajectories, followed by support vector machine stacking. At 13 h before cardiac arrest, the model reached an AUROC of 0.85 in MIMIC-IV, with external validation AUROCs of 0.81 in eICU-CRD and 0.945 in the National Taiwan University Hospital cohort. This work demonstrated the value of combining static clinical information with temporal physiological patterns, but did not use clinical narrative text or radiology information.

Recent work has also addressed hemodynamic deterioration in cardiovascular ICU patients. Gao et al. [23] developed machine learning models using MIMIC-IV and eICU-CRD with bidirectional external validation. The final random forest model achieved an AUROC of 0.841 when trained on MIMIC-IV and tested on eICU-CRD, and an AUROC of 0.852 when trained on eICU-CRD and tested on MIMIC-IV. The model outperformed traditional clinical scores such as SOFA [24] and APACHE II [25], and its risk stratification showed increasing mortality from 0.8% in the very low-risk group to 84.2% in the very high-risk group. This study is closely related to cardiovascular ICU risk prediction and provides strong evidence for the value of external validation; however, it relied mainly on structured clinical variables and did not include radiology reports.

Beyond building models, other studies have examined the deployment of early warning systems, showing that such systems should be measured not only in terms of discrimination statistics but in how they affect clinical workflows and outcomes. Verma et al. [26] tested CHARTwatch, which is a real-time machine learning early warning system implemented in a general internal medicine ward. During the intervention period, non-palliative deaths in the hospital were less than during the pre-intervention period among general internal medicine patients, with a relative risk adjusted for 0.74. In high-risk patients, non-palliative deaths dropped from 10.3% to 7.1%, although the confidence interval crossed one. These results suggest that predictive models may be clinically useful when combined with alerts and care pathways, but also show that deployment and workflow integration are central to real-world impact.

A related line of work has explored the integration of clinical text with structured data. Teoh et al. [27] studied heart failure prediction using MIMIC-IV and MIMIC-IV-ED, combining structured clinical variables with BioClinicalBERT embeddings from admission notes. Their best model was random forest, which achieved an accuracy of 0.8994 and an AUROC of 0.9659. In addition, their ablation analysis suggested that text embeddings led to improved performance. This study supports the idea that clinical narratives can add useful information beyond structured variables; however, the task was heart failure classification rather than early ICU deterioration prediction, and the text source was admission notes rather than temporally filtered radiology reports.

In summary, while the studies reviewed above show important progress in ICU deterioration prediction, a specific gap remains. Prior studies have mainly focused on mortality, cardiac arrest, qSOFA-based deterioration, or hemodynamic deterioration in specific ICU populations, and have typically relied on structured variables, vital sign trajectories, ECG-derived features, or clinical text, either alone or in limited combinations. However, the reviewed studies do not directly evaluate the same three-modality setting used in our paper, which combines enriched first-24-h structured clinical features, 24-h vital sign time series, and timestamp-filtered radiology report embeddings for early prediction of a composite ICU deterioration endpoint defined by vasopressor initiation and/or early death. Therefore, the methodological contribution of the present study is to integrate these complementary modalities within a leakage-controlled multimodal prediction pipeline, followed by systematic comparison against unimodal, early fusion, late fusion, and ablation baselines.

## 3. Methodology

This section describes the methodology used for early cardiovascular deterioration prediction. The presentation follows a progressive structure: first, the prediction problem is defined; then, each of the modalities (structured clinical variables, vital sign time series, and radiology reports) is described independently; finally, the three modality-specific representations are combined into a multimodal neural fusion model.

### 3.1. Problem Formulation

The objective is to predict early cardiovascular deterioration for each ICU stay using information available during the early ICU period. The prediction target is a binary composite endpoint. An ICU stay is labeled as positive if vasopressor initiation and/or early death occurs during the defined outcome window. This endpoint is broader than mortality prediction alone because it also includes patients who require hemodynamic support but survive.

Let $v_{i}$ denote vasopressor initiation and $m_{i}$ denote early death for ICU stay *i*. The binary target label is defined as

(1) $$y_{i}=I\left(v_{i}=1\vee m_{i}=1\right),$$

where I(·) is the indicator function. The model estimates the probability of ICU stay *i* belonging to the positive class.

### 3.2. Structured Clinical Representation

Structured clinical data provide information about the early clinical state of the patient. These variables include demographic information, vital signs, and laboratory measurements extracted during the first 24 h of ICU stay. Two structured representations were used: a structured baseline representation for unimodal models, and an enriched clinical representation, referred to as rich24, for the proposed multimodal model.

#### 3.2.1. Structured Baseline Variables

The structured baseline representation was used to evaluate the predictive information available from structured clinical data alone. It included demographic variables and selected early clinical variables such as vital signs and laboratory measurements, as summarized in Table 1.

#### 3.2.2. Enriched Clinical Representation

The proposed multimodal model used an enriched first-24-h structured representation called rich24. Instead of using only one value per clinical variable, rich24 summarizes the early evolution of selected vital signs and laboratory measurements during the first 24 h of an ICU stay.

For each selected variable, the extracted descriptors included the number of available measurements, first value, last value, minimum, maximum, mean, standard deviation, temporal trend, and missingness indicators. The temporal trend was computed as the difference between the last and first available values. These descriptors were concatenated across all selected variables to form the final rich24 structured feature vector.

**Figure 1:**
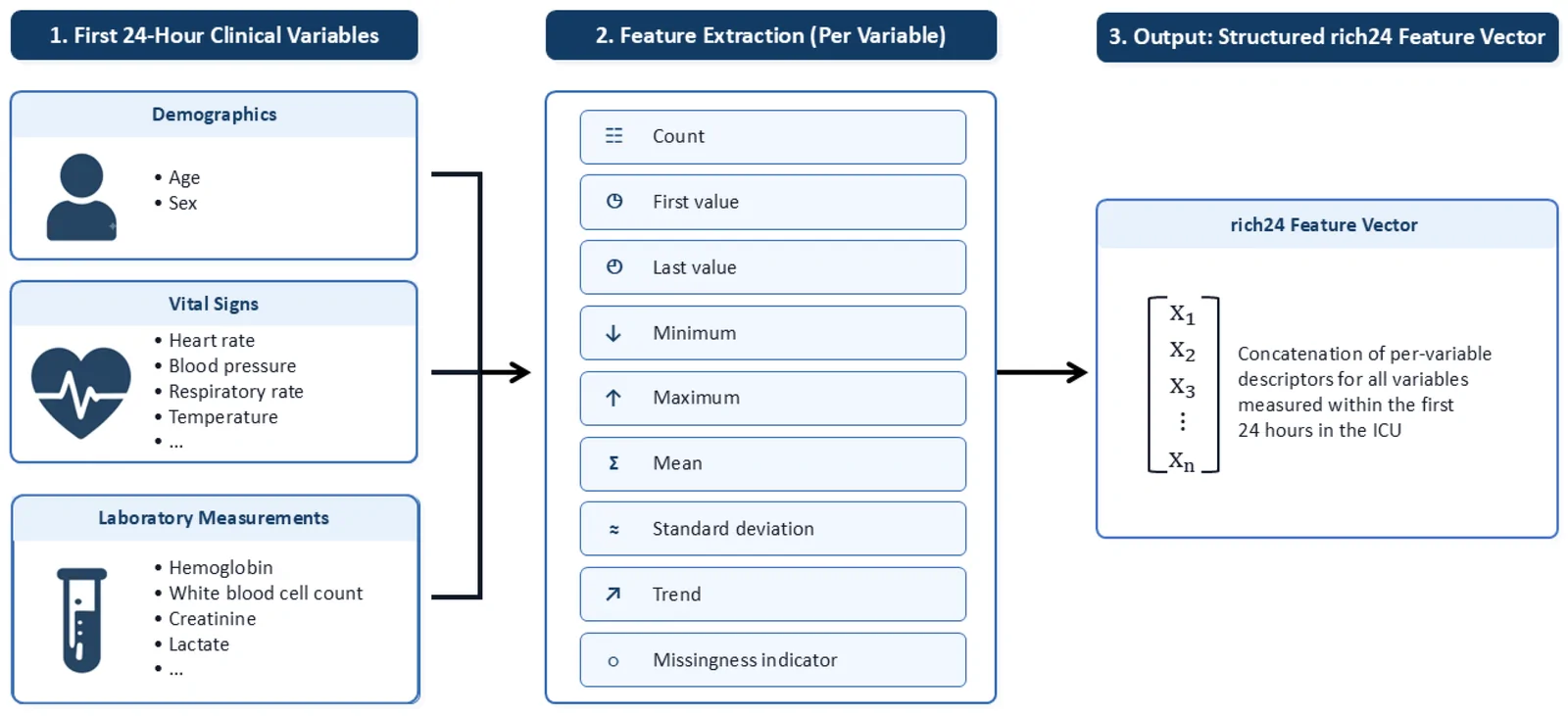
Construction of the rich24 structured clinical representation. First-24-h demographic, vital sign, and laboratory variables were summarized using per-variable descriptors, including count, first value, last value, minimum, maximum, mean, standard deviation, temporal trend, and missingness indicators. These descriptors were concatenated to form the rich24 structured feature vector used by the structured branch of MultiCardioNet.

The structured representation for ICU stay *i* is encoded as

(2) $$h_{i}^{S}=f_{S}\left(x_{i}^{S}\right),$$

where $x_{i}^{S}$ is the structured clinical input and $f_{S}\left(\cdot \right)$ denotes the structured encoder. Figure 1 illustrates the construction of the rich24 representation from first-24-h clinical variables and per-variable summary descriptors.

### 3.3. Vital Sign Time Series Representation

The time series modality represents the temporal evolution of vital signs during the first 24 h of ICU stay. Each ICU stay was represented as a 24-h sequence with six physiological channels: heart rate, systolic blood pressure, diastolic blood pressure, mean blood pressure, respiratory rate, and peripheral oxygen saturation.

For the summary-based time series baseline, the sequence was summarized using descriptive statistics, including mean, standard deviation, minimum, maximum, first value, last value, and temporal trend. This produced a fixed-length representation that was used by the summary-based XGBoost model.

For the proposed multimodal neural model, the full 24-h sequence was processed using a transformer-based time series encoder [8]. The input sequence was projected into a latent dimension, positional information was added, and transformer encoder blocks were used to model temporal relationships across the observation window. A pooling operation was then used to obtain a fixed-length temporal representation.

**Figure 2:**
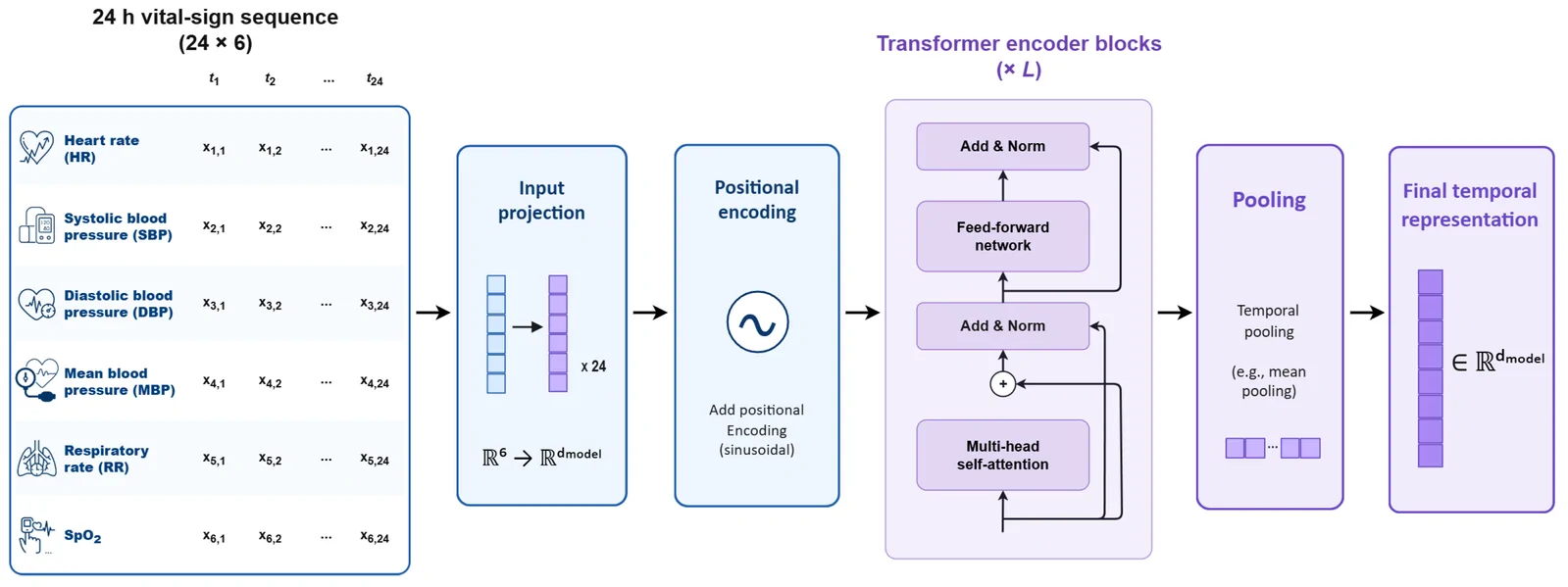
Transformer-based encoder for 24-h vital sign time series. The input sequence contains six physiological channels aggregated into hourly bins, followed by input projection, positional encoding, transformer encoder blocks, pooling, and a final temporal representation.

The time series representation for ICU stay *i* is encoded as

(3) $$h_{i}^{T}=f_{T}\left(x_{i}^{T}\right),$$

where $x_{i}^{T}$ is the 24-h vital sign sequence and $f_{T}\left(\cdot \right)$ denotes the transformer-based time series encoder. Figure 2 summarizes the structure of this branch, from the 24-h input sequence to the final temporal representation.

### 3.4. Radiology Text Representation

Radiology reports were used as the text modality. These reports may contain clinical interpretation of cardiopulmonary findings that are not fully represented in structured variables or vital sign time series. Only early radiology reports aligned with the prediction setting were used.

ICU stays without eligible timestamp-filtered radiology reports were retained in the cohort. For these stays, the radiology branch received a fixed-dimensional missing text representation, ensuring that all ICU stays had the same radiology input dimensionality. This strategy avoided excluding patients solely because eligible radiology text was unavailable. Because radiology availability may reflect clinical workflow, documentation patterns, temporal filtering, or patient severity, it was evaluated separately as a sensitivity analysis.

Discharge summaries were excluded from the final modeling pipeline because they are written after hospitalization and may contain information about diagnoses, treatments, complications, and outcomes. Excluding discharge summaries helps to reduce the risk of outcome leakage and preserves an early prediction setting.

#### Radiology Text Encodings

Two radiology text representations were evaluated. For the radiology TF-IDF baseline, eligible timestamp-filtered radiology reports were represented using TF-IDF features [28]. For the CXR-BERT-based radiology baselines and the proposed multimodal model, eligible radiology reports were represented using CXR-BERT-specialized embeddings.

CXR-BERT-specialized [13] is a radiology-oriented language model built on the BERT architecture [12], and is designed to encode chest radiology text into dense semantic representations. In this study, eligible radiology reports were passed through the CXR-BERT-specialized encoder, and the CLS-token representation was used as the report-level embedding. The representation was extracted using a maximum sequence length of 512 tokens and projected to 256 dimensions using TruncatedSVD. The CXR-BERT encoder was not fine-tuned during downstream model training.

The radiology representation for ICU stay *i* is encoded as

(4) $$h_{i}^{R}=f_{R}\left(e_{i}^{R}\right),$$

where $e_{i}^{R}$ is the projected CXR-BERT-specialized embedding extracted from eligible timestamp-filtered radiology reports and $f_{R}\left(\cdot \right)$ denotes the radiology text encoder.

### 3.5. Unimodal Baselines

Unimodal baselines were trained to evaluate the contribution of each data type independently. For structured clinical data, logistic regression, random forest [29], and XGBoost [30] were evaluated using demographic, vital sign, and laboratory variables.

For the time series modality, XGBoost was trained on summary statistics extracted from the 24-h vital sign sequence. In addition, LSTM [6], TCN [7], and transformer [8] models used the full 24-h sequence directly, allowing the summary-based representation and sequence-based neural encoders to be evaluated separately.

For radiology text, TF-IDF features and CXR-BERT-specialized embeddings were evaluated using eligible timestamp-filtered radiology reports. The radiology baselines included a TF-IDF representation, a CXR-BERT embedding-based radiology model, and an XGBoost classifier trained on CXR-BERT radiology embeddings.

These unimodal models provide reference points for assessing the predictive contribution of each individual data source before evaluating multimodal fusion models.

### 3.6. Multimodal Neural Fusion

The proposed model combines the three modality-specific representations described above. The structured branch encodes the rich24 clinical feature vector, the time series branch encodes the 24-h vital sign sequence using a transformer encoder, and the radiology branch encodes CXR-BERT-specialized radiology embeddings using a dense neural branch.

The three branch outputs are concatenated as follows:

(5) $$h_{i}=\left[h_{i}^{S};h_{i}^{T};h_{i}^{R}\right].$$

The final prediction is obtained using a multilayer perceptron classifier:

(6) $${\hat{p}}_{i}=\sigma \left(W_{2}\;\delta \left(W_{1}h_{i}+b_{1}\right)+b_{2}\right),$$

where ${\hat{p}}_{i}$ is the predicted probability of cardiovascular deterioration, δ(·) is the nonlinear activation function, σ(·) is the sigmoid function, and $W_{1}$, $W_{2}$, $b_{1}$, and $b_{2}$ are learnable parameters.

The novelty of MultiCardioNet lies in its task-specific integration of three complementary ICU data sources within a leakage-controlled prediction framework rather than in the introduction of a new fusion formula. These modalities include enriched first-24-h structured clinical features, 24-h vital sign time series, and timestamp-filtered radiology report embeddings. The studies reviewed in this manuscript address related ICU deterioration tasks using structured variables, vital sign trajectories, ECG-derived features, or clinical text, but do not directly evaluate this same three-modality setting for early prediction of the composite ICU deterioration endpoint. The use of modality-specific encoders followed by neural fusion provides a unified framework in which each data source can be represented according to its structure before joint risk estimation. This architecture also supports direct comparison with unimodal baselines, early-fusion XGBoost models, late-fusion multimodal baselines, and modality ablation models.

**Figure 3:**
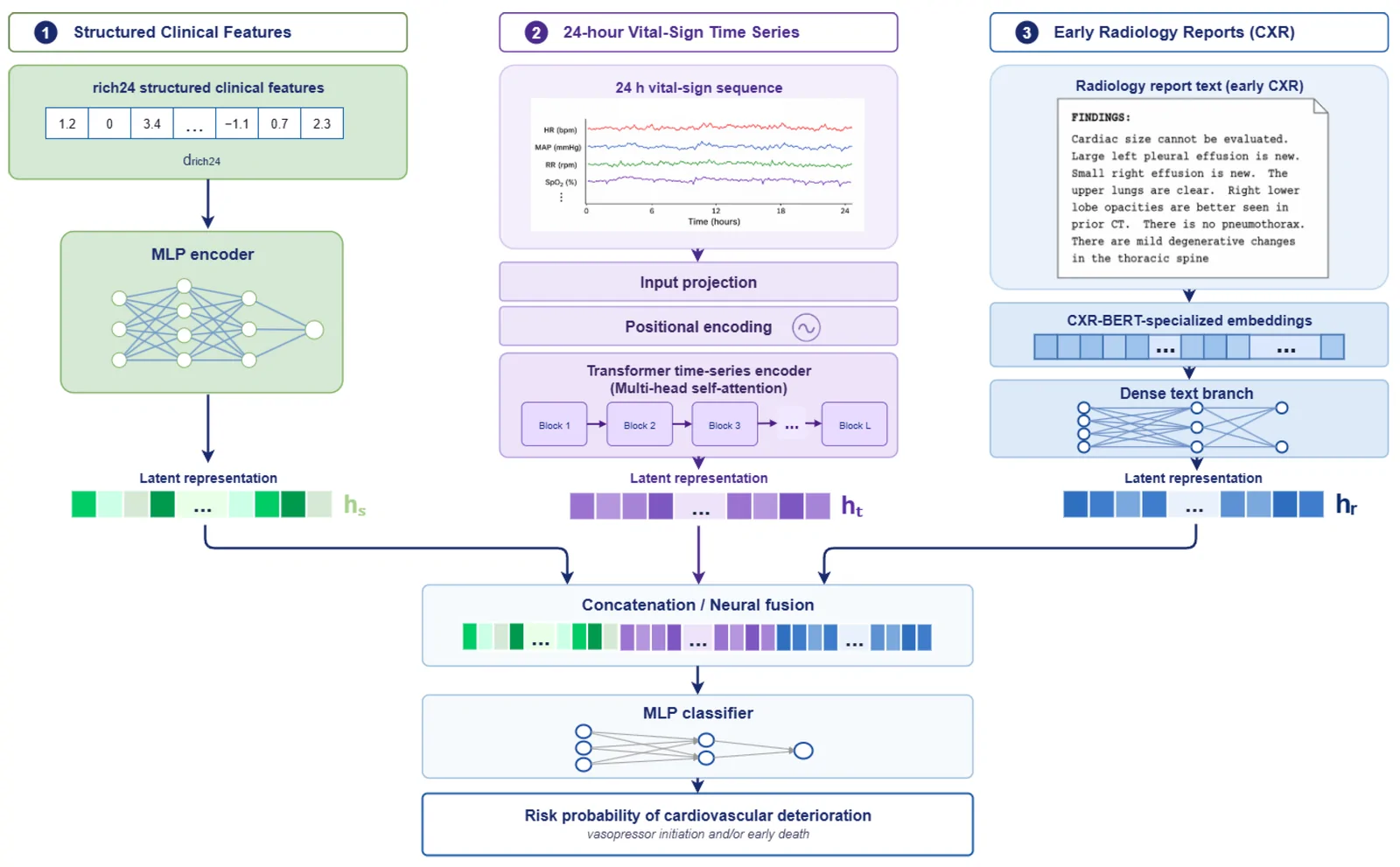
Overall architecture of MultiCardioNet. The model combines rich24 structured clinical features, transformer-based 24-h vital sign encoding, and CXR-BERT-specialized radiology embeddings. The three latent representations are concatenated and passed through an MLP classifier to estimate cardiovascular deterioration risk.

Figure 3 summarizes the complete MultiCardioNet architecture. The model processes structured clinical features, vital sign time series, and early radiology reports in separate branches before concatenating the latent representations for final risk prediction.

The configuration of the proposed multimodal neural fusion model is summarized in Table 2.

### 3.7. Decision Threshold

The model outputs a continuous probability score between 0 and 1. To obtain a binary prediction, a decision threshold is applied to the predicted probability. Threshold selection was performed using the validation set. The threshold that maximized validation F1-score was selected and then applied once to the held-out test set for final evaluation.

### 3.8. Summary of the Proposed Method

The methodology first evaluates each modality independently using unimodal baselines, then combines the three modalities in a neural fusion model. The final model uses rich24 structured clinical features, a transformer-based vital sign time series encoder, and CXR-BERT-specialized radiology embeddings to estimate early cardiovascular deterioration risk.

## 4. Materials and Experimental Setup

This section describes the data sources, cohort construction, data preprocessing, model configurations, training procedure, loss function, and evaluation metrics used in this study. The experimental setup focuses on the three modalities used in MultiCardioNet: structured clinical variables, 24-h vital sign time series, and radiology reports represented using CXR-BERT-specialized embeddings.

### 4.1. Data Sources

This study used data from the Medical Information Mart for Intensive Care IV (MIMIC-IV) ecosystem [1]. Structured clinical information was extracted from the MIMIC-IV hospital and ICU modules. Radiology reports were extracted from MIMIC-IV-Note and used as the text modality. To preserve an early prediction setting, the final model used only early ICU information. The data sources and corresponding model modalities are summarized in Table 3.

The detailed content extracted from these sources is summarized in Table 4. This table describes each modality by its fields, clinical meaning, and format.

### 4.2. Cohort Construction

The cohort was constructed at the level of ICU stay. Only adult patients were included. To avoid repeated ICU stays from the same individual, only the first ICU stay was retained for each patient. ICU stays shorter than 24 h were excluded because the proposed model uses a 24-h observation window for structured and temporal feature extraction.

For each ICU stay, structured clinical features and vital sign time series features were extracted from the first 24 h. Radiology reports available during the early prediction period were aligned to the corresponding ICU stay. ICU stays without available radiology reports were retained in the cohort and represented using a missing text embedding strategy.

The final aligned cohort contained 54,551 ICU stays, including 19,665 positive cases for the cardiovascular deterioration endpoint and 34,886 negative cases, corresponding to a positive rate of 36.05%.

### 4.3. Temporal Prediction Design

The prediction task was formulated using a landmark-based temporal design. The observation window covered the first 24 h after ICU admission. During this interval, structured clinical variables, vital sign time series data, and radiology reports were collected as model inputs. A risk prediction was generated at the 24-h landmark.

**Figure 4:**
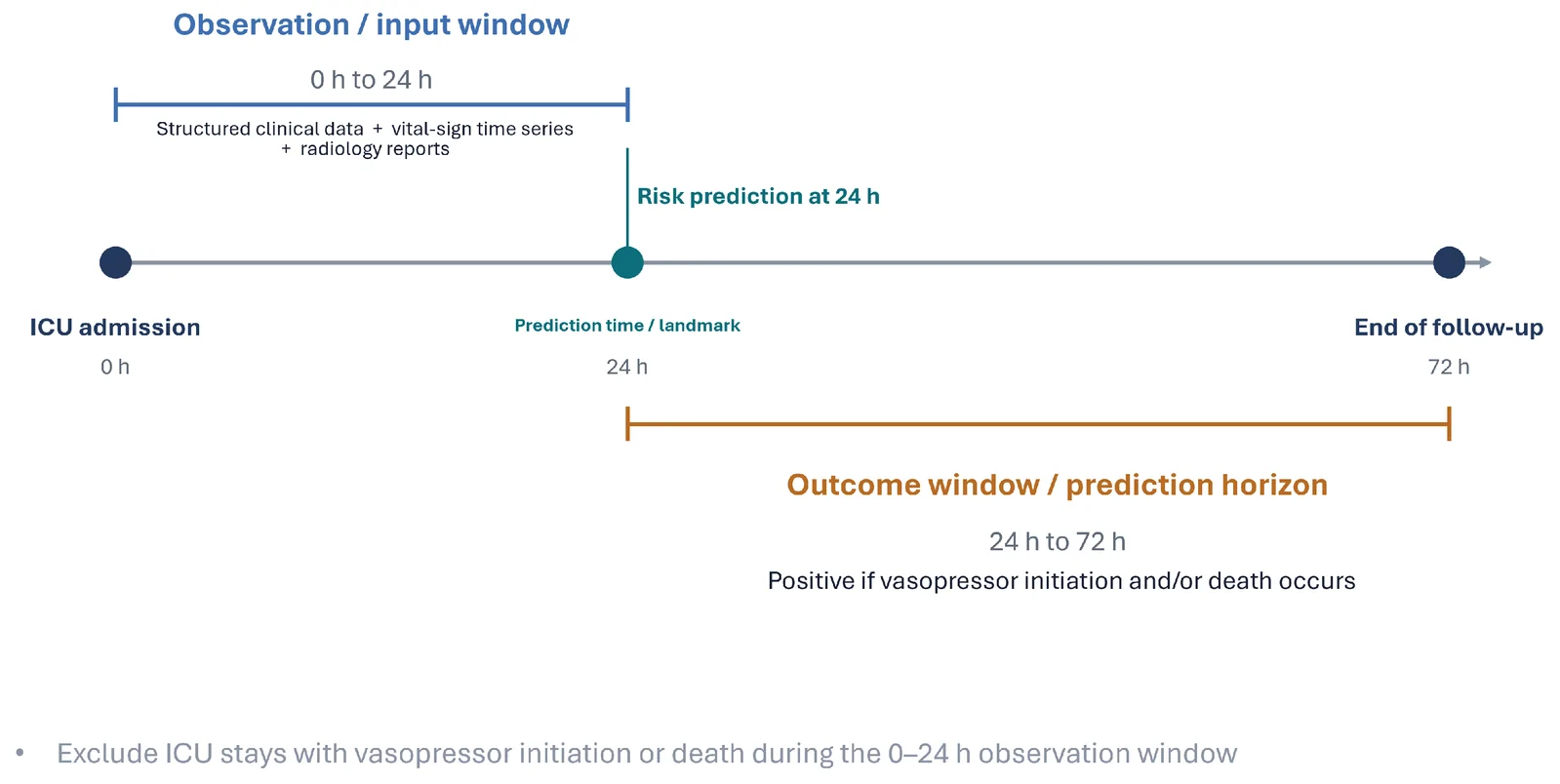
Temporal prediction design used in this study. Model inputs were collected during the first 24 h after ICU admission, a prediction was generated at the 24-h landmark, and the outcome was assessed from 24 to 72 h.

The outcome window extended from 24 to 72 h after ICU admission. An ICU stay was labeled positive if vasopressor initiation and/or death occurred during this interval. ICU stays with vasopressor initiation or death during the first 24 h were excluded from the analysis in order to preserve temporal separation between model inputs and outcome occurrence. Figure 4 illustrates the temporal prediction design used in this study.

### 4.4. Outcome Definition

The prediction target was a binary composite cardiovascular deterioration endpoint. An ICU stay was labeled positive if vasopressor initiation and/or early death occurred during the 24–72 h outcome window after ICU admission. This endpoint is broader than mortality alone because it also captures patients who required hemodynamic support but survived.

To describe the composition of the composite endpoint, positive cases were decomposed into vasopressor-only cases, death-only cases, and cases with both vasopressor initiation and death. Among the 19,665 positive cases, 17,988 were vasopressor-only cases, 669 were death-only cases, and 1008 included both vasopressor initiation and death (Table 5).

### 4.5. Train, Validation, and Test Splits

The cohort was divided into training, validation, and test sets using stratified sampling. The cohort was constructed at the ICU stay level, and only the first ICU stay was retained for each patient. As a result, each patient contributed at most one ICU stay to the final aligned cohort. The split was then performed at the patient/ICU-stay level, so the same patient could not appear in more than one split.

The training set was used to fit model parameters. The validation set was used for model selection, calibration fitting, and threshold selection. The held-out test set was used only once for final evaluation. The split used a fixed random seed of 42 and preserved the outcome prevalence across the three sets. The final split included 34,912 ICU stays for training, 8728 for validation, and 10,911 for testing, corresponding to approximately 64%, 16%, and 20% of the cohort, respectively. The split distribution is summarized in Table 6.

### 4.6. Data Preprocessing

#### 4.6.1. Structured Clinical Variables

Structured clinical variables were extracted from demographic tables, ICU chart events, laboratory events, and medication records. The structured baseline representation included age, sex, first available vital signs, and first available laboratory measurements during the early observation period.

For the proposed multimodal model, the enriched rich24 representation was used as described in Section 3.2.2. Continuous features were standardized using statistics computed from the training set.

Structured variables and rich24 descriptors were computed using measurements available during the 0–24 h observation window only.

#### 4.6.2. Vital Sign Time Series Preprocessing

The time series modality was constructed from the first 24 h of vital sign measurements. Six physiological channels were included: heart rate, systolic blood pressure, diastolic blood pressure, mean blood pressure, respiratory rate, and oxygen saturation. Each ICU stay was represented as an hourly sequence with 24 time steps and 6 channels.

Measurements were aggregated into hourly bins. When multiple measurements were available within the same hour, their average was used. Missing values were handled by forward filling within the patient sequence when possible, followed by median filling. Time series channels were standardized using statistics computed from the training set.

For the summary-based time series XGBoost baseline, the 24-h sequence was converted into summary statistics. For each of the six channels, the mean, standard deviation, minimum, maximum, first value, last value, and trend were extracted, producing 42 time series summary features. For the neural time series models, the full 24-h sequence was used directly.

All vital sign time series values were extracted only from the 0–24 h observation window, before the 24-h prediction landmark.

#### 4.6.3. Radiology Text Preprocessing

Radiology reports were extracted from MIMIC-IV-Note and aligned with ICU stays. To reduce the risk of outcome leakage, reports were filtered using report-level timestamps before text embedding generation. Report time was defined using report storage/finalization time. Chart time was considered as a fallback during preprocessing if storage/finalization time was unavailable; however, in the retained radiology report table used for modeling, all reports had available storage/finalization times. For positive cases, radiology reports were retained only if the report time occurred before the first recorded outcome event or before the 24-h observation cutoff, whichever occurred first. For negative cases, radiology reports were retained only if the report time occurred within the first 24 h after ICU admission.

When more than one eligible radiology report was available for an ICU stay, the report texts were concatenated into a single text field. ICU stays without eligible timestamp-filtered radiology reports were retained in the cohort and represented using a fixed-dimensional missing text representation. This strategy allowed all ICU stays to keep the same radiology input dimensionality and avoided excluding patients solely because eligible radiology text was unavailable. Because radiology availability itself may reflect clinical workflow, documentation patterns, temporal filtering, or patient severity, radiology availability was evaluated separately in a sensitivity analysis.

Two radiology text representations were evaluated. For the TF-IDF radiology baseline, eligible radiology reports were represented using TF-IDF features. For the CXR-BERT radiology baseline and the proposed multimodal model, eligible radiology reports were encoded using CXR-BERT-specialized embeddings. The CLS-token representation was extracted using a maximum sequence length of 512 tokens and projected to 256 dimensions using TruncatedSVD.

### 4.7. Experimental Models

The experimental comparison included unimodal models, multimodal fusion baselines, proposed-architecture ablations, and the final MultiCardioNet model. The unimodal models were used to evaluate the predictive contribution of structured clinical variables, vital sign time series, and radiology text independently. The multimodal fusion baselines were used to compare early fusion and late fusion strategies. The ablation models were used to evaluate the contribution of different modality combinations within the proposed neural architecture.

The structured logistic regression model used class weighting. Structured RF and XGBoost were used to evaluate nonlinear structured baselines. The time series models evaluated both engineered summary features and neural sequence encoders. The radiology models evaluated sparse TF-IDF features and dense CXR-BERT-specialized embeddings. The early-fusion XGBoost baselines used concatenated multimodal features, while the late-fusion baselines combined modality-specific predictions at the output level. The proposed MultiCardioNet model used a structured MLP branch, a transformer-based time series branch, a CXR-BERT-specialized radiology branch, and an MLP fusion classifier. The experimental models evaluated in this study are summarized in Table 7.

### 4.8. Loss Function

The neural models were trained as binary classifiers using binary cross-entropy with logits. Because the outcome was imbalanced, a positive-class weight was used during training. For a sample *i*, the model produces a logit $s_{i}$ and predicted probability ${\hat{p}}_{i}=\sigma \left(s_{i}\right)=1/\left(1+e^{-s_{i}}\right)$.

The weighted binary cross-entropy loss is

(7) $$L=-\frac{1}{N}\sum_{i=1}^{N}\left[w_{+}y_{i}\log\left({\hat{p}}_{i}\right)+\left(1-y_{i}\right)\log\left(1-{\hat{p}}_{i}\right)\right],$$

where $w_{+}$ is the positive-class weight. In the experiments, $w_{+}$ was computed from the training set as

(8) $$w_{+}=\frac{N_{-}}{N_{+}},$$

where $N_{+}$ and $N_{-}$ denote the number of positive and negative training samples, respectively. For logistic regression baselines, class weighting was also applied so that the positive and negative classes contributed more evenly to the training objective.

### 4.9. Evaluation Metrics

Model performance was evaluated using AUROC, AUPRC, accuracy, precision, recall, and F1-score. Since the positive endpoint was imbalanced, AUPRC was considered an important ranking metric [31]. F1-score was used to summarize the trade-off between precision and recall at a selected decision threshold.

Precision was defined as

(9) $$\mathrm{Precision}=\frac{TP}{TP+FP},$$

where TP denotes true positives and FP denotes false positives.

Recall was defined as

(10) $$\mathrm{Recall}=\frac{TP}{TP+FN},$$

where FN denotes false negatives.

The F1-score was computed as

(11) $$F1=\frac{2\times \mathrm{Precision}\times \mathrm{Recall}}{\mathrm{Precision}+\mathrm{Recall}}.$$

Accuracy was computed as

(12) $$\mathrm{Accuracy}=\frac{TP+TN}{TP+TN+FP+FN},$$

where TN denotes true negatives.

For each model, probabilities were converted into binary predictions using a decision threshold τ:

(13) $${\hat{y}}_{i}=\left\{\begin{array}{cc} 1, & \mathrm{if}\;{\hat{p}}_{i}\geq \tau ,\\ 0, & \mathrm{otherwise}. \end{array}\right.$$

A threshold sweep was performed on the validation set, and the threshold maximizing the validation F1-score was selected:

(14) $$\tau^{*}=\arg\max_{\tau }F1_{\mathrm{val}}\left(\tau \right).$$

The selected threshold was then applied to the test set. This procedure enabled threshold selection without using test set labels.

### 4.10. Statistical Analysis

Uncertainty estimates were computed using ICU stay-level bootstrap resampling of the held-out test set. ICU stays were resampled with replacement, and model performance was recomputed for each bootstrap sample. For AUROC, AUPRC, accuracy, precision, recall, specificity, negative predictive value, and F1-score, the 95% confidence intervals were calculated using the 2.5th and 97.5th percentiles of the bootstrap distribution.

To compare MultiCardioNet with the strongest baseline, paired bootstrap resampling was performed using the same resampled ICU stays for both models. The strongest baseline was defined as the best-performing non-MultiCardioNet model in the main model comparison table. In this study, this corresponded to the structured + time series + radiology XGBoost early fusion model, which combined structured clinical variables, time series summary features, and radiology text features before XGBoost classification. Absolute differences in performance metrics were calculated as MultiCardioNet minus the strongest baseline for each bootstrap sample. Empirical two-sided paired bootstrap *p*-values were computed from the distribution of paired differences.

For this statistical comparison, probability outputs were calibrated using Platt scaling fitted on validation predictions only. Decision thresholds were then selected on validation calibrated probabilities by maximizing validation F1-score and applied once to the held-out test set. Because calibration and threshold selection can change binary predictions, threshold-dependent metrics from the bootstrap comparison were interpreted separately from the original model comparison operating points.

Calibration was evaluated on the held-out test set using Brier score, expected calibration error, maximum calibration error, calibration intercept, calibration slope, and calibration curves. Calibration curves were generated using ten quantile-based risk bins. For each bin, the mean predicted risk was compared with the observed event rate. Calibration intercept and slope were estimated by logistic regression of the observed outcome on the logit-transformed predicted risk, with perfect calibration corresponding to an intercept of 0 and a slope of 1. Post hoc Platt calibration was fitted using validation set predictions only, then applied once to the held-out test set. Uncalibrated and Platt-calibrated probabilities were compared to assess whether calibration improved the reliability of the predicted risk estimates.

Threshold selection was performed using validation-set predictions only. The held-out test set was used only once for final evaluation of the selected operating points. Because a single F1-optimized threshold may not reflect all clinical priorities, two operating points were evaluated: a balanced operating point selected by maximizing the validation F1-score, and a lower-alert operating point selected to reduce the alert burden while maintaining clinically interpretable performance. For each operating point, the sensitivity, specificity, positive predictive value, negative predictive value, accuracy, F1-score, and alert burden were reported on the held-out test set.

Decision curve analysis was performed to evaluate net benefit across a range of threshold probabilities. Net benefit was calculated for MultiCardioNet and the structured + time series + radiology XGBoost baseline, then compared with treat-all and treat-none strategies.

To assess training stability, the final MultiCardioNet architecture was evaluated across three random seeds. The same data split, preprocessing pipeline, model architecture, and hyperparameter setting were retained, while model initialization and stochastic training components were varied across seeds. Performance was summarized as mean ± standard deviation across the three runs.

Interpretability was assessed using permutation-based analysis on the held-out test set. Modality groups, structured features, time series channels, and time series time blocks were shuffled, and the decrease in AUROC and AUPRC was used to estimate their contribution to model performance. Permutation analysis was repeated across five repeats. Because permutation importance measures changes in model performance after disrupting an input, it was interpreted as model behavior analysis rather than as causal evidence.

Because the final radiology branch used dense CXR-BERT embeddings, individual radiology terms could not be directly interpreted from the embedding space. Therefore, a TF-IDF surrogate analysis was performed to provide a term-level approximation of the radiology signal. The surrogate model used TF-IDF radiology terms and ridge regression to approximate the final MultiCardioNet risk score among ICU stays with eligible radiology text. It was trained on validation-set ICU stays for which text was available, then evaluated on test-set ICU stays for which text was available. This analysis was interpreted as a surrogate explanation of the CXR-BERT-based radiology signal.

Subgroup analysis was performed on the held-out test set. Performance was evaluated across age group, sex, radiology availability group, ICU care unit, and race group. ICU care unit and race information were obtained by linking held-out test set predictions to MIMIC-IV ICU stay and admission metadata using ICU stay and admission identifiers. The first care unit, last care unit, and race were available for all test cases, while a separate ethnicity field was not available. Subgroup analyses were interpreted descriptively because subgroup size, outcome prevalence, and data availability differed across groups.

### 4.11. Experimental Comparison Strategy

The comparison strategy was designed to evaluate the progression from unimodal prediction to multimodal fusion. First, structured clinical data, vital sign time series, and radiology text were evaluated independently using modality-specific unimodal models. Second, early-fusion XGBoost baselines were evaluated using two-modality and three-modality feature combinations. Third, late-fusion averaging and stacking were evaluated as prediction-level multimodal fusion baselines. Finally, MultiCardioNet and its proposed-architecture ablations were evaluated to assess the contribution of different modality combinations within the neural fusion framework.

### 4.12. Implementation and Reproducibility Details

The study used MIMIC-IV version 3.1 and MIMIC-IV-Note version 2.2 radiology reports. Adult first ICU stays were identified from ICU stay tables and linked to demographic, admission, laboratory, vital sign, medication, and radiology note data using patient and admission identifiers.

The SQL extraction logic selected the first ICU stay per patient, restricted structured variables and vital sign time series inputs to the first 24 h after ICU admission, and excluded ICU stays with vasopressor initiation or death during the 0–24 h observation window. The prediction task was defined at the 24-h landmark, and the outcome window extended from 24 to 72 h after ICU admission.

The outcome was defined as cardiovascular deterioration during the 24–72 h outcome window. Vasopressor initiation was identified from medication administration records using vasopressor definitions corresponding to norepinephrine/Levophed, epinephrine/adrenaline, phenylephrine/Neo-Synephrine, vasopressin, dopamine, and dobutamine. Early death was defined using the recorded death time when death occurred during the 24–72 h outcome window.

Radiology reports were filtered before embedding generation using report-level timestamps. Report time was defined using report storage/finalization time. Chart time was considered as a fallback during preprocessing if storage/finalization time was unavailable; however, in the retained radiology report table used for modeling, all retained reports had available storage/finalization times. For positive cases, reports were retained only if the report time occurred before the first recorded outcome event or before the 24-h observation cutoff, whichever occurred first. For negative cases, reports were retained only if the report time occurred within the first 24 h after ICU admission. Reports not satisfying these criteria were excluded before radiology embedding extraction.

Radiology text was encoded using the microsoft/BiomedVLP-CXR-BERT-specialized checkpoint. The CLS token representation was extracted using a maximum sequence length of 512 tokens. The CXR-BERT encoder was frozen and was not fine-tuned during downstream model training. The resulting embeddings were projected to 256 dimensions using TruncatedSVD before being passed to the radiology branch.

The final MultiCardioNet architecture used a structured MLP branch, a transformer-based time series branch, a radiology text branch, and an MLP fusion classifier. The main model used a hidden dimension of 256, branch dimension of 128, time series embedding dimension of 128, four attention heads, two transformer layers, and dropout of 0.30. Training used AdamW optimization with a learning rate of $1\times 10^{-4}$, weight decay of $1\times 10^{-4}$, batch size of 192, and binary cross-entropy with logits loss using positive-class weighting. Early stopping was based on validation AUPRC with a patience of 15 epochs, and training was run for a maximum of 100 epochs.

The data split seed was 42. The final architecture was evaluated across model seeds 42, 7, and 123 to assess training stability, and the primary reported MultiCardioNet model used seed 7. The implementation used Python with PyTorch, Hugging Face Transformers, scikit-learn, XGBoost, NumPy, and pandas. Key implementation and reproducibility details for the final MultiCardioNet model are summarized in Table 8.

### 4.13. Computational Considerations

The computational cost of MultiCardioNet comes from three components: structured feature processing, time series encoding, and radiology text representation. The structured branch operates on the fixed-dimensional rich24 feature vector, and has a lower computational cost than the sequence and text components. The time series branch uses a transformer encoder over 24 hourly time steps and six vital sign channels. Therefore, the self-attention cost remains limited by the short sequence length used in this study.

The radiology text component is the most computationally demanding part of the pipeline; however, because the CXR-BERT-specialized encoder was frozen and radiology embeddings were precomputed before downstream model training, the final MultiCardioNet model was trained using fixed 256-dimensional radiology embeddings rather than end-to-end fine-tuning of the full language model. This reduced the downstream training cost, required additional preprocessing time and storage for radiology embeddings. Overall, although the architecture increases computational cost compared to unimodal models, this cost is mainly associated with radiology embedding extraction and multimodal preprocessing rather than with the final fusion classifier.

## 5. Results

This section presents the experimental results of the unimodal baselines and MultiCardioNet. The results are reported on the held-out test set. The main objective was to evaluate whether combining structured clinical information, 24-h vital sign dynamics, and CXR-BERT-specialized radiology embeddings improves early cardiovascular deterioration prediction compared with models that use each modality independently.

### 5.1. Unimodal Baseline Results

Several unimodal baselines were evaluated in order to measure the predictive contribution of each modality independently. For structured clinical data, we evaluated logistic regression, random forest, and XGBoost models. For the time series modality, summary-based XGBoost and neural sequence models were evaluated using the 24-h vital sign sequence. The neural sequence models included LSTM, TCN, and transformer encoders. For radiology text, TF-IDF features and CXR-BERT-specialized embeddings were evaluated.

Among the structured-only models, XGBoost achieved the highest performance with an AUROC of 0.8329, AUPRC of 0.7193, and F1-score of 0.6902. Structured random forest achieved an AUROC of 0.8220, AUPRC of 0.7170, and F1-score of 0.6802. Structured logistic regression achieved lower performance, with an AUROC of 0.7603, AUPRC of 0.6258, and F1-score of 0.6323. These results indicate that the nonlinear structured models captured more predictive information from early clinical variables than the linear structured baseline.

Among the time series-only models, the summary-based XGBoost model achieved an AUROC of 0.8334, AUPRC of 0.7255, and F1-score of 0.6959. The time series transformer achieved a similar AUROC of 0.8263 and an AUPRC of 0.7228, while the LSTM achieved an AUROC of 0.8227 and an AUPRC of 0.7156. The performance of the TCN model was lower in this setting.

For radiology text, the TF-IDF model achieved an AUROC of 0.8038, AUPRC of 0.7115, and F1-score of 0.6738. The CXR-BERT-specialized radiology model achieved an AUROC of 0.7852, AUPRC of 0.7017, and F1-score of 0.6511. The radiology CXR-BERT embeddings + XGBoost baseline achieved lower discrimination, with an AUROC of 0.6897 and an AUPRC of 0.4848.

### 5.2. MultiCardioNet Performance

MultiCardioNet combined three inputs: enriched structured clinical features, 24-h vital sign time series encoded with a transformer, and timestamp-filtered radiology reports represented using CXR-BERT-specialized embeddings. On the held-out test set, the final model achieved an AUROC of 0.9014, AUPRC of 0.8481, accuracy of 0.8256, and F1-score of 0.7723 at the validation-selected operating threshold. The corresponding precision was 0.7294 and recall was 0.8205.

### 5.3. Model Comparison

Table 9 compares representative unimodal, early fusion, late fusion, and MultiCardioNet models on the held-out test set. The models are grouped by modality type to make the comparison clearer.

Among the unimodal baselines, the structured XGBoost model achieved the highest structured-only performance, followed by structured random forest and structured logistic regression. For time series-only models, summary-based XGBoost and the transformer encoder achieved the strongest performance. For radiology-only models, the TF-IDF baseline performed higher than the CXR-BERT-based radiology baselines in this setting.

The two-modality early-fusion XGBoost baselines improved over their corresponding single-modality baselines. The strongest early-fusion baseline used all three modalities and achieved an AUROC of 0.8905, AUPRC of 0.8070, and F1-score of 0.7600. Late-fusion average and late-fusion stacking were evaluated as multimodal S + T + R fusion baselines combining modality-specific predictions at the output level. These baselines achieved AUROC values of 0.8787 and 0.8786, respectively.

Because the radiology-only TF-IDF baseline performed better than the radiology-only CXR-BERT baseline, TF-IDF was also evaluated within the full multimodal setting. The optimized TF-IDF multimodal model achieved an AUROC of 0.8961, AUPRC of 0.8359, accuracy of 0.8303, precision of 0.7612, recall of 0.7709, and F1-score of 0.7660. The final CXR-BERT-based MultiCardioNet achieved an AUROC of 0.9014, AUPRC of 0.8481, accuracy of 0.8256, precision of 0.7294, recall of 0.8205, and F1-score of 0.7723. Although the TF-IDF multimodal model achieved slightly higher accuracy and precision, the CXR-BERT-based model achieved higher AUROC, AUPRC, recall, and F1-score; therefore, CXR-BERT embeddings were retained in the final multimodal model.

MultiCardioNet achieved higher AUROC, AUPRC, accuracy, recall, and F1-score results than the evaluated comparison models on the held-out internal test set. These results suggest that the three modalities provide complementary information when using this dataset.

### 5.4. Uncertainty Estimates, Statistical Comparison, and Seed Stability

ICU stay-level bootstrap resampling was used to estimate 95% confidence intervals for the main test set performance metrics. MultiCardioNet was compared with the strongest non-MultiCardioNet baseline from Table 9. This baseline was the structured + time series + radiology XGBoost early fusion model, which used all three modalities through feature-level concatenation followed by XGBoost classification. The comparison used Platt-calibrated probabilities and validation-selected calibrated thresholds. The bootstrap confidence intervals and paired comparison with the strongest baseline are summarized in Table 10.

The bootstrap analysis shows that MultiCardioNet achieved higher AUROC and AUPRC than the strongest baseline. Recall and negative predictive value also favored MultiCardioNet. Differences in accuracy, precision, and specificity were small and not statistically significant at the 0.05 level, while the F1-score difference was small and borderline. Overall, the main advantage of MultiCardioNet over the strongest baseline was most evident in threshold-independent discrimination and precision–recall performance.

The final MultiCardioNet architecture was also evaluated across three random seeds to assess training stability. The same data split, preprocessing pipeline, architecture, and hyperparameter setting were retained across runs. The seed-stability results are summarized in Table 11.

MultiCardioNet showed limited variation across random seeds for AUROC, AUPRC, and F1-score. The test AUROC ranged from 0.8988 to 0.9014, the test AUPRC ranged from 0.8455 to 0.8490, and the test F1-score ranged from 0.7693 to 0.7723, suggesting that the main performance pattern was not dependent on a single model initialization.

### 5.5. Calibration Analysis

Calibration was assessed on the held-out test set by comparing uncalibrated MultiCardioNet probabilities with Platt-calibrated probabilities. Platt calibration was fitted using validation set predictions only, then applied to the held-out test set. Calibration metrics for uncalibrated and Platt-calibrated probabilities are summarized in Table 12.

**Figure 5:**
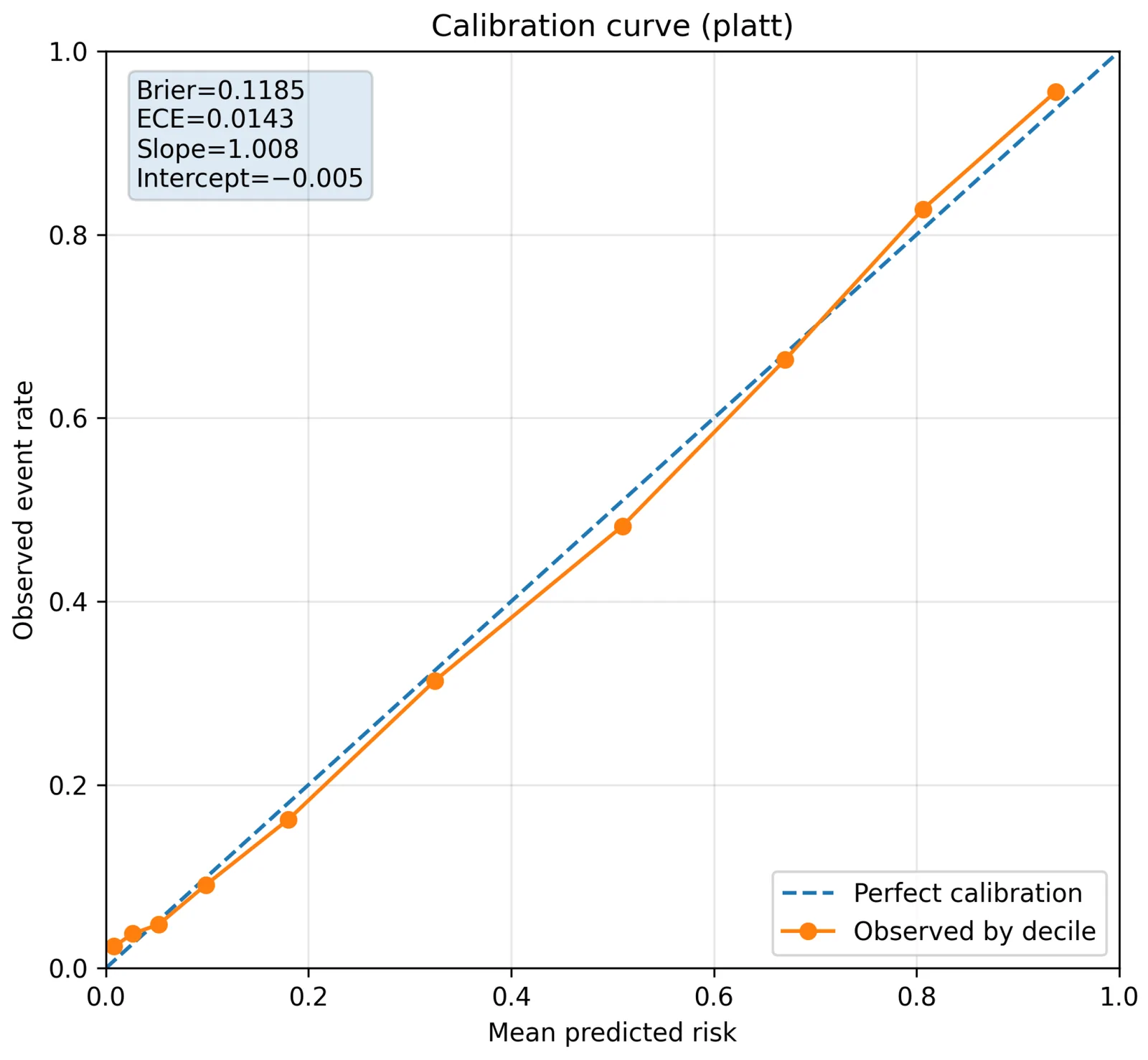
Calibration curve for Platt-calibrated MultiCardioNet probabilities on the held-out test set. The points show quantile-based risk bins, with the dashed diagonal line representing perfect calibration.

Platt calibration improved the Brier score, ECE, and MCE compared to the uncalibrated model. The calibration intercept moved closer to 0 and the calibration slope moved closer to 1, indicating better agreement between predicted and observed risks. The mean Platt-calibrated predicted risk was 0.3614, close to the observed test set event rate of 0.3605. The calibration curve for Platt-calibrated MultiCardioNet probabilities is shown in Figure 5.

The observed and predicted risks by decile are shown in Table 13. The Platt-calibrated probabilities followed the observed event rates across the risk spectrum, with the largest absolute calibration error of 0.0276.

### 5.6. Multimodal Ablation Analysis

The ablation analysis evaluated two-modality and three-modality configurations within the proposed neural architecture. This analysis was used to assess how performance changed when different modality combinations were used. The multimodal ablation results are summarized in Table 14.

The full structured + time series + radiology text configuration achieved the highest AUROC, AUPRC, accuracy, and F1-score among the evaluated ablation configurations. The structured + radiology text configuration was the strongest two-modality setting, while the full model further improved performance by incorporating vital sign dynamics.

### 5.7. Radiology Availability and Missing Text Analysis

Because eligible timestamp-filtered radiology reports were not available for all ICU stays, the performance of MultiCardioNet was evaluated separately for ICU stays with and without eligible radiology text. This analysis was performed in order to assess whether model performance was concentrated within one subgroup for which radiology text was available. Performance stratified by eligible radiology-text availability is reported in Table 15.

Performance differed between ICU stays with and without eligible timestamp-filtered radiology text. MultiCardioNet maintained discrimination in both subgroups, with AUROC values above 0.82. The subgroup without eligible radiology text showed higher AUPRC and F1-score. This pattern indicates that radiology availability defines clinically and operationally non-equivalent patient subsets. Radiology availability may reflect clinical workflow, documentation patterns, imaging use, temporal filtering, and patient case mix. Therefore, these subgroup results are interpreted descriptively rather than as a direct comparison between exchangeable patient groups.

A radiology-availability-only model was also evaluated, using only a binary indicator for the presence of eligible timestamp-filtered radiology text. This model did not use radiology report content. The predictive value of radiology-text availability alone is reported in Table 16.

Although radiology availability carried proxy information, its predictive value was substantially lower than the full multimodal model. Thus, radiology availability alone does not explain the full performance of MultiCardioNet.

### 5.8. Endpoint Component Analysis

To further examine the composite endpoint, we evaluated model performance separately for vasopressor-only cases, death-only cases, and cases with both vasopressor initiation and death. The primary composite threshold was selected on validation predictions only, then applied one time to the held-out test set. At this threshold of 0.41, the full composite endpoint included 3933 positive test cases and achieved an AUROC of 0.9014, AUPRC of 0.8481, recall of 0.8159, and F1-score of 0.7710.

For vasopressor-only cases versus negative cases, the model achieved an AUROC of 0.9039, AUPRC of 0.8406, recall of 0.8224, and F1-score of 0.7640 at the same composite threshold. For the less frequent death-only and vasopressor-plus-death components, component-specific thresholds were selected on validation predictions and applied once to the held-out test set. The death-only comparison achieved an AUROC of 0.7773, AUPRC of 0.1105, recall of 0.2237, and F1-score of 0.1481. For cases with both vasopressor initiation and death, the model achieved an AUROC of 0.9491, AUPRC of 0.4847, recall of 0.4829, and F1-score of 0.4737. The component-level results are summarized in Table 17.

The component-level analysis indicates that the composite endpoint was mainly associated with vasopressor-related cases. The death-only subgroup was small and showed lower recall and F1-score than the full composite endpoint. Therefore, the overall model performance should be interpreted in relation to the composition of the composite endpoint.

### 5.9. Threshold Analysis and Decision Curve Analysis

Because MultiCardioNet outputs a continuous probability score for cardiovascular deterioration, a decision threshold is required when converting this score into a binary alert. Threshold selection was performed using validation-set predictions only, and the selected operating points were then applied once to the held-out test set.

Because maximizing F1-score may not reflect all clinical priorities, two operating points were evaluated. The first was a balanced operating point selected by maximizing validation F1-score. The second was a lower-alert operating point selected to reduce alert burden while maintaining higher precision and specificity. Table 18 summarizes sensitivity, specificity, positive predictive value, negative predictive value, accuracy, F1-score, and alert burden for these operating points.

The balanced operating point provided higher recall and F1-score, identifying 81.59% of positive cases with an alert burden of 40.24%. The lower-alert operating point reduced the alert burden to 29.89% and increased PPV and specificity, but this came with lower recall. These results show the expected clinical trade-off between identifying more at-risk patients and reducing the number of alerts.

**Figure 6:**
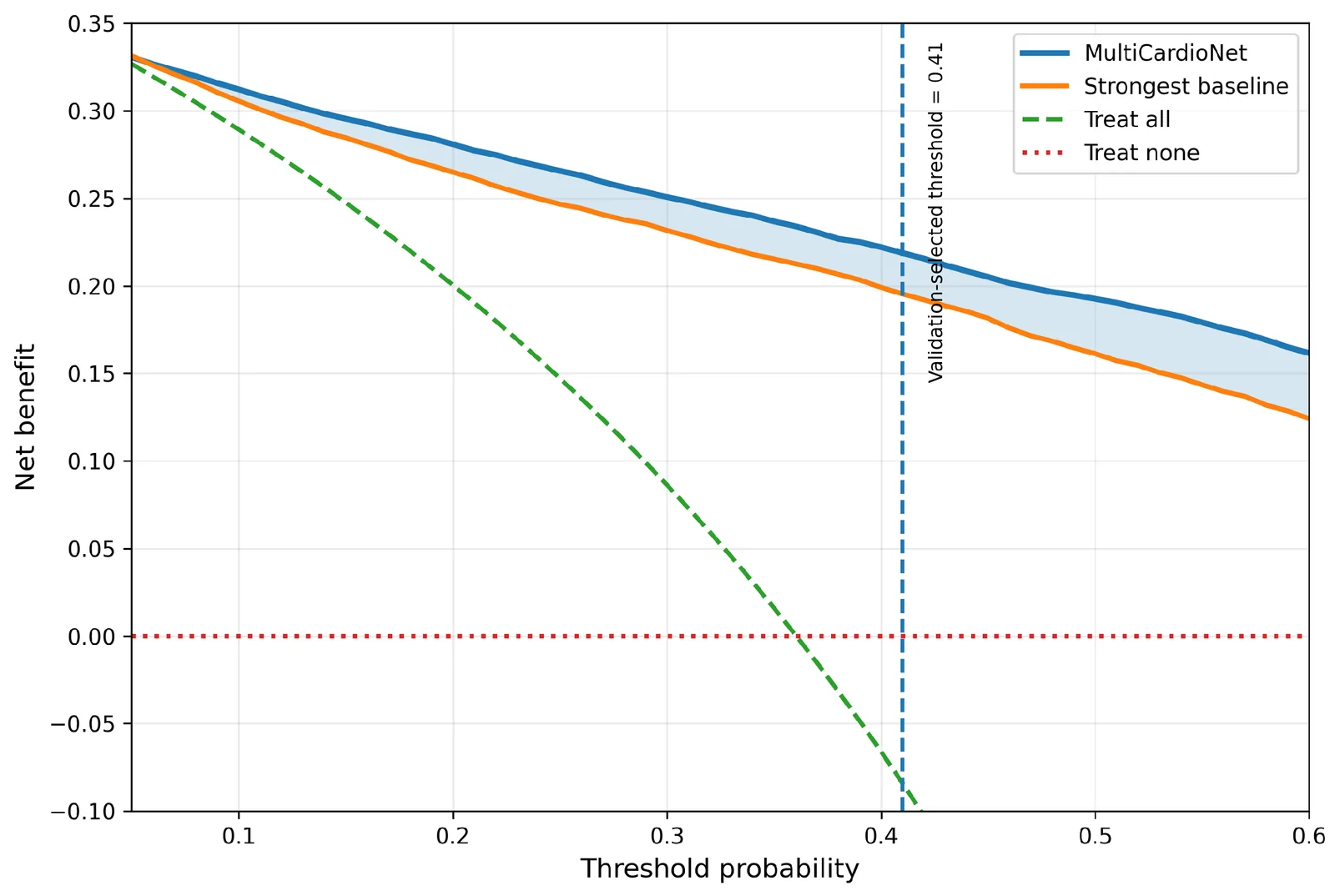
Decision curve analysis on the held-out test set. Net benefit is shown across threshold probabilities. MultiCardioNet is compared with the strongest baseline as well as with the treat-all and treat-none strategies.

Decision curve analysis was performed to evaluate model utility across a range of threshold probabilities rather than at a single operating point. MultiCardioNet showed higher net benefit than the strongest baseline across the evaluated threshold range on the held-out test set. The decision curve is shown in Figure 6.

### 5.10. Interpretability and Subgroup Analysis

Permutation-based analysis was used to assess the contribution of model inputs on the held-out test set. Importance was quantified as the decrease in AUROC and AUPRC after shuffling each modality or feature group. This analysis was interpreted as a description of model behavior rather than as evidence of causal effects. Permutation-based modality importance is summarized in Table 19.

The largest performance decrease occurred after perturbing the structured feature branch, followed by the radiology text branch and the time series branch. This indicates that all three modalities contribute to model performance, with the strongest dependence on structured clinical features in this analysis. The top structured features ranked by permutation-based AUPRC decrease are shown in Table 20.

Among structured variables, the most influential feature was minimum systolic blood pressure during the observation window. Several missingness and measurement count features also contributed, suggesting that both measured values and measurement patterns influenced model predictions. Permutation-based time series importance is summarized in Table 21.

The time series permutation analysis showed that performance was most affected by perturbing blood pressure channels, especially diastolic BP, systolic BP, and mean BP. Across time blocks, the first 4 h and the later 16–24 h period showed the largest decreases in AUPRC. These results suggest that both early physiological status and later changes during the 24-h observation window contributed to prediction.

For radiology text, a TF-IDF surrogate model was trained to approximate final MultiCardioNet risk among ICU stays with eligible radiology reports. The surrogate was trained on ICU stays for which validation text was available, and was evaluated on test ICU stays for which text was available. The surrogate achieved an $R^{2}$ of 0.2263 and an RMSE of 0.2628 for approximating final model probability, indicating that TF-IDF radiology terms captured part but not all of the risk signal from the CXR-BERT-based model.

The radiology surrogate analysis identified terms related to clinical instability, circulatory support, catheter/device descriptions, and cardiopulmonary status. These terms should be interpreted cautiously, since the analysis approximates the final model probability using a sparse TF-IDF surrogate and does not directly explain the CXR-BERT encoder. Representative radiology terms associated with higher and lower surrogate-predicted MultiCardioNet risk are shown in Table 22.

Subgroup performance was evaluated across age, sex, and radiology availability groups, as summarized in Table 23.

Subgroup results were interpreted descriptively because subgroup size, outcome prevalence, and data availability differed across groups. Performance was broadly similar across age and sex groups for AUROC, although AUPRC and threshold-dependent metrics differed with outcome prevalence. The radiology availability groups showed larger differences, consistent with the missing text analysis showing that eligible radiology text was not a neutral data characteristic. Additional subgroup results by ICU care unit and simplified race group are reported in Table 24.

ICU care unit and race information were available for all 10,911 held-out test cases after linkage with MIMIC-IV ICU stay and admission metadata. A separate ethnicity field was not available. Across major first care unit groups, AUROC values were generally between 0.8592 and 0.8969, excluding smaller neuro-specific groups. AUPRC and threshold-dependent metrics varied more strongly across care units, reflecting differences in endpoint prevalence and case mix. Across simplified race groups, AUROC ranged from 0.8705 to 0.9280.

## 6. Discussion

### 6.1. Overall Results Interpretation

The results indicate that MultiCardioNet achieved strong performance on the internal test set for prediction of the composite ICU deterioration endpoint. Compared with the evaluated unimodal and multimodal baselines, the model achieved higher AUROC, AUPRC, accuracy, recall, and F1-score, suggesting that the three modalities provided complementary information in this dataset.

The component-level analysis showed that model performance was mainly associated with vasopressor-related endpoint components. This finding is consistent with the study definition of cardiovascular deterioration, which includes vasopressor initiation and/or early death. Vasopressor initiation reflects clinically important circulatory support, but may also depend on differences in patient severity and ICU management practices. Death-only cases were less well identified, likely because they represented a small subgroup of positive cases. These results indicate that the reported performance should be interpreted in relation to the makeup of the composite endpoint.

The baseline and ablation analyses showed that performance improved when complementary modalities were combined. Early-fusion XGBoost with structured variables, time series features, and radiology embeddings achieved strong performance; late-fusion averaging and stacking also performed competitively. Within the proposed neural architecture, the full structured + time series + radiology text configuration achieved the highest AUROC, AUPRC, accuracy, and F1-score among the ablation configurations. These findings support the value of combining structured clinical variables, vital sign dynamics, and radiology report representations within a unified multimodal prediction framework.

The uncertainty analysis further supported the model comparison. ICU stay-level bootstrap confidence intervals showed that MultiCardioNet had higher AUROC and AUPRC than the strongest early-fusion XGBoost baseline. The paired bootstrap analysis also favored MultiCardioNet for recall and negative predictive value, while differences in accuracy, precision, and specificity were small. The seed stability analysis showed limited variation across three random seeds, suggesting that the main performance pattern was not dependent on a single model initialization.

The radiology baselines suggested that early radiology reports contained predictive information related to the endpoint. However, the radiology-only models remained below the multimodal models, indicating that radiology-derived information was more informative when combined with structured clinical variables and physiological time series data. This supports the use of radiology text as a complementary modality rather than as a standalone source for this task.

As an additional assessment of generalizability, we performed an external validation experiment on eICU-CRD using the structured and time series components of the model. Full multimodal external validation was not performed because the radiology text modality and final feature space could not be directly harmonized between MIMIC-IV/MIMIC-IV-Note and eICU-CRD. The structured + time series model achieved an AUROC of 0.8715, AUPRC of 0.7946, and F1-score of 0.7329 on the internal MIMIC-IV test set. On eICU-CRD, the same non-text model achieved an AUROC of 0.7151, AUPRC of 0.4186, and F1-score of 0.4151. This difference should be interpreted within the context of the reduced modality set, smaller harmonized structured feature space, and differences between the two ICU databases. Additional external validation with harmonized multimodal data is needed before drawing stronger conclusions about generalizability.

Overall, the findings suggest that the proposed framework can combine structured clinical variables, vital sign dynamics, and radiology report representations for cardiovascular deterioration prediction in the internal MIMIC-IV setting. Structured clinical variables describe the patient’s early clinical state, while vital sign sequences provide short-term physiological dynamics and radiology reports add clinical interpretation from imaging. The external validation experiment also indicates that further testing across harmonized independent datasets remains necessary.

### 6.2. Implications of the Multimodal Architecture

The architecture was designed to process each modality according to its structure. The structured branch uses the rich24 representation to summarize first-24-h clinical measurements and their short-term changes. The time series branch models the 24-h vital sign sequence using a transformer-based encoder, while the text branch uses CXR-BERT-specialized embeddings extracted from early radiology reports. These three representations are then combined in a neural fusion classifier.

This design is clinically plausible because the composite endpoint may be associated with information distributed across different clinical sources, including early structured measurements, vital sign dynamics, and radiology report content. A patient may show abnormal laboratory values, unstable vital signs, or radiological signs of cardiopulmonary disease, and a single modality may capture only part of this process. By combining modalities, the model can use evidence distributed across multiple clinical sources rather than depending on one data type alone.

The results suggest that modality-specific representation learning followed by neural fusion is useful for this prediction task. The model learns separate representations for structured, temporal, and textual information before combining them for final risk estimation. This design preserves the clinical meaning of each modality while allowing the model to use interactions between early clinical measurements, physiological dynamics, and radiology-derived text features.

### 6.3. Contribution of Baselines and Ablation Analyses

The baseline and ablation analyses help to clarify the contribution of each data type and fusion strategy. The structured baselines showed that early demographic, vital sign, and laboratory features contain important predictive information. The structured XGBoost and random forest models performed higher than structured logistic regression, suggesting that nonlinear relationships among early clinical variables were relevant for cardiovascular deterioration prediction.

The time series baselines showed that vital sign dynamics also contribute to the prediction task. Summary-based XGBoost and the transformer encoder achieved the strongest time series-only performance among the evaluated models, suggesting that both engineered summaries and sequence-based modeling can capture useful information from the 24-h vital sign window.

The radiology baselines showed that early radiology reports contain predictive information related to the endpoint. The TF-IDF radiology baseline performed higher than the CXR-BERT-based radiology baselines in the unimodal setting. This indicates that radiology-derived information was useful, but not sufficient by itself to reach the performance of multimodal models.

The early-fusion XGBoost models showed that combining structured variables, time series summary features, and radiology embeddings can improve performance over the corresponding single-modality XGBoost models. Late-fusion averaging and stacking also performed competitively as multimodal S + T + R prediction-level fusion baselines, although they remained below the strongest early-fusion XGBoost baseline and the final MultiCardioNet model.

The proposed-architecture ablations showed that the full structured + time series + radiology text model achieved the highest AUROC, AUPRC, accuracy, and F1-score among the evaluated ablation configurations. The structured + radiology text configuration was the strongest two-modality setting, while the full model further improved performance by incorporating vital sign dynamics. These results suggest that MultiCardioNet benefits from integrating complementary clinical views: structured variables describe the early clinical state, time series data capture short-term physiological changes, and radiology text adds imaging-based clinical context. This multimodal design supports prediction using evidence distributed across different data sources rather than relying on one modality alone.

### 6.4. Leakage Control and Use of Radiology Reports

A major design decision in this study was the exclusion of discharge summaries from the final modeling pipeline. Discharge summaries are written after hospitalization and often include retrospective information about diagnoses, treatments, complications, and outcomes; thus, their use in an early prediction setting could introduce outcome leakage and inflate model performance.

Radiology reports were retained because they can provide clinical information closer to the early ICU period, but were filtered using report-level timestamps before embedding generation. Report time was defined using report storage/finalization time. Chart time was considered as a fallback during preprocessing if storage/finalization time was unavailable; however, in the retained radiology report table used for modeling, all retained reports had available storage/finalization times. For positive cases, only reports finalized before the first recorded outcome event or before the 24-h observation cutoff were retained. For negative cases, only reports within the first 24 h after ICU admission were retained. Reports not satisfying these rules were excluded before CXR-BERT embedding generation.

Structured variables and vital sign time series data were also restricted to the 0–24 h observation window. Together with the exclusion of ICU stays with vasopressor initiation or death during the first 24 h, this design was used to maintain temporal separation between model inputs and the outcome window.

To verify radiology-related leakage control, we examined the retained radiology report timestamp table used for modeling. All 37,270 retained radiology reports had storage/finalization times available through storetime; therefore, no retained report required chart time to be used as a fallback in the final radiology input table. A pipeline cutoff audit also showed no retained reports after the radiology input cutoff. This supports the temporal filtering of the retained radiology reports, although the analysis remains retrospective and depends on the accuracy of recorded report and event timestamps.

Radiology report availability was not uniform across the cohort after timestamp filtering. ICU stays without eligible radiology text were retained using the missing-text embedding strategy, which avoided removing stays from the analysis solely because eligible radiology text was unavailable.

The radiology availability analysis showed that eligible radiology text was not a neutral data characteristic. MultiCardioNet showed different performance in ICU stays with and without eligible timestamp-filtered radiology reports. This subgroup difference should not be interpreted as evidence that the absence of eligible radiology text improves prediction; rather, it indicates that radiology availability defines clinically and operationally different patient subsets. Radiology availability may reflect documentation patterns, imaging use, temporal filtering, illness trajectory, or patient case mix.

The radiology-availability-only model showed that while availability carried proxy information, its predictive value was substantially lower than the full multimodal model. This suggests that model performance was not explained only by the presence or absence of eligible radiology text. Nevertheless, radiology availability should be considered when interpreting the radiology branch, as the text modality may reflect both the content of radiology reports and contextual information about patient care.

### 6.5. Clinical Interpretation and Threshold Selection

The model outputs a continuous probability score for cardiovascular deterioration. Because probability calibration is important for risk prediction, calibration was evaluated in addition to discrimination. Platt calibration fitted on validation predictions improved Brier score, ECE, and calibration slope/intercept on the held-out test set. Therefore, the calibrated output provides a better estimate of observed risk than the uncalibrated neural network probability output in the internal test set.

The threshold analysis showed that the model can be evaluated at different operating points depending on the intended use. The balanced validation-selected threshold provided higher recall and F1-score, while the lower-alert operating point increased PPV and specificity while reducing the proportion of ICU stays flagged as high risk. This illustrates that threshold selection should be based on the clinical objective, for example, prioritizing sensitivity for early screening or prioritizing PPV and alert burden for more selective alerts.

The decision curve analysis provided an additional assessment of net benefit across risk thresholds. MultiCardioNet showed higher net benefit than the strongest baseline across the evaluated threshold range. However, these operating points should be interpreted as retrospective evaluation results rather than deployment recommendations. Prospective evaluation would be needed to define clinically acceptable thresholds in real-world workflows.

### 6.6. Interpretability, Subgroup Performance, and Fairness Considerations

The interpretability analyses provide an initial view of model behavior. Permutation-based analysis identified inputs where disruption reduced discrimination, while the radiology TF-IDF surrogate analysis identified terms for which sparse text representation approximated part of the final CXR-BERT-based risk signal. These analyses suggest that the model used information from structured clinical variables, vital sign dynamics, and radiology text; however, these findings should not be interpreted as causal explanations.

The structured feature permutation analysis highlighted minimum systolic blood pressure and several measurement count or missingness features, suggesting that both physiological values and patterns of clinical measurement contribute to prediction. The time series permutation analysis showed that some channels and time blocks were more influential than others, suggesting that the model used temporal information from the 24-h observation window.

The radiology surrogate analysis identified terms related to shock, arrest, sepsis, hypotension, vascular access devices, and cardiopulmonary status as being associated with higher surrogate-predicted risk. This supports the interpretation that radiology text may contain clinically relevant information. However, several terms also reflected device or care context, so the radiology branch should be interpreted as a radiology report signal rather than a purely imaging-derived signal.

The subgroup analysis showed that model performance varied across age group, sex, radiology availability, ICU care unit, and race group. Performance was broadly similar across age and sex groups for AUROC, while larger differences were observed for radiology availability and some ICU care unit groups. The ICU care unit and race analyses showed broad preservation of discrimination across the evaluated groups, although AUPRC and threshold-dependent metrics varied with endpoint prevalence and case mix. Therefore, the subgroup analysis should be interpreted as a descriptive safety assessment rather than a complete fairness evaluation.

Radiology availability was an important source of subgroup heterogeneity. This is consistent with the analyses of missing text and radiology availability, which showed that eligible radiology text was not a neutral data characteristic. These findings indicate that data availability and documentation patterns should be considered when interpreting the radiology branch.

### 6.7. Limitations

Several limitations should be considered when interpreting the results. The composite endpoint was not equally represented across its components. Most positive cases involved vasopressor initiation, while death-only and vasopressor-plus-death cases were less frequent. Although component-level analysis was performed, the model was optimized for the full composite endpoint and not separately optimized for rare death-only events. Vasopressor initiation reflects clinically important circulatory support, but can also vary with patient severity, treatment decisions, local protocols, and ICU management practices. Therefore, the reported performance should be interpreted in relation to the composition of the composite endpoint.

The study was based on retrospective EHR data. Several steps were used to reduce outcome leakage, including exclusion of ICU stays with vasopressor initiation or death during the 0–24 h observation window, restriction of structured and time series inputs to the first 24 h, and timestamp filtering of radiology reports before embedding generation. Review of the retained radiology report table showed that all retained radiology reports had available storage/finalization times, and no retained report required use of the chart time fallback in the final radiology input table. However, retrospective temporal alignment still depends on the reliability of recorded event times and report timestamps, which limits the ability to fully reproduce real-time data availability from retrospective records.

Calibration and model evaluation were performed mainly in the internal MIMIC-IV setting. Platt calibration improved the Brier score, expected calibration error, and calibration slope/intercept on the held-out test set; however, since calibration performance may change across institutions, patient populations, documentation workflows, and clinical practice patterns, the calibrated probabilities should be interpreted as internally calibrated risk estimates rather than deployment-ready clinical probabilities.

Full multimodal external validation was not performed. External validation was restricted to the structured + time series components, as the final MultiCardioNet checkpoint used 256 structured input features, a 24 × 6 vital sign time series input, and a 256-dimensional CXR-BERT/SVD radiology embedding while the harmonized eICU-CRD dataset provided 68 structured features, the same 24 × 6 vital sign time series format, and no directly comparable radiology text representation. Therefore, applying the final full checkpoint directly to eICU-CRD would not provide a valid external validation of the complete multimodal model.

Eligible timestamp-filtered radiology reports were not available for all ICU stays. ICU stays without eligible radiology text were retained using the missing-text representation, but radiology availability itself carried proxy information and defined non-equivalent patient subsets. The radiology-availability-only model showed lower performance than the full multimodal model, indicating that availability alone does not explain the full model performance. Nevertheless, missingness and report availability should be considered when interpreting the radiology branch.

Subgroup and fairness analysis included age group, sex, radiology availability, ICU care unit, and race group; however, subgroup sizes, endpoint prevalence, and data availability differed across groups and a separate ethnicity field was not available. Therefore, these subgroup results should be interpreted as a descriptive safety analysis rather than a complete fairness evaluation.

The proposed model used concatenation-based intermediate fusion of modality-specific representations. This design supports direct comparison with unimodal, early fusion, late fusion, and ablation baselines; however, other fusion mechanisms may capture cross-modal interactions differently. In addition, the radiology text branch required precomputed CXR-BERT embeddings, which add to the preprocessing time and storage requirements. Runtime, memory use, and computational efficiency were not evaluated as primary outcomes in this study.

### 6.8. Future Work Directions

Several research directions could build upon this work. First, full multimodal external validation using harmonized independent ICU datasets remains important. In the present study, external validation was limited to the structured and time series components, since the radiology text modality and final feature space could not be directly harmonized between MIMIC-IV/MIMIC-IV-Note and eICU-CRD. A harmonized multimodal cohort across independent ICU datasets would allow for evaluation of the complete MultiCardioNet architecture outside of the internal MIMIC-IV setting.

Prospective evaluation is also needed in order to assess model behavior under real-time data availability and clinical workflow constraints. Such evaluation would help to determine whether the model outputs remain stable when only prospectively available structured variables, vital signs, and finalized radiology reports are used. External and prospective recalibration would also be needed before interpreting model outputs as clinically reliable probabilities in deployment.

Another direction is the evaluation of alternative multimodal fusion strategies. The present model uses concatenation-based intermediate fusion of modality-specific representations. Future studies could compare this approach with cross-modal attention, gated fusion, mixture-of-experts fusion, or other mechanisms designed to model interactions between structured variables, physiological time series, and radiology report embeddings.

Handling missing data is also important for multimodal ICU prediction. Not all ICU stays had eligible radiology reports after temporal filtering, so future models could include dedicated missing-modality learning strategies to better handle missing text or varying data availability. Computationally efficient approaches should also be explored, especially for reducing the preprocessing and storage costs associated with radiology text embeddings.

Further explainability and subgroup evaluation are also needed. Methods such as feature attribution, attention visualization, modality-specific analysis, and more complete subgroup calibration could help to assess whether the model relies on clinically meaningful signals and whether performance remains consistent across clinically relevant patient groups.

Finally, ECG waveform modeling and echocardiography-based representations could be explored as additional cardiac-specific modalities when sufficient data and appropriate temporal alignment are available.

## 7. Conclusions

In this work, we have presented MultiCardioNet, a multimodal deep learning framework for early prediction of a composite ICU deterioration endpoint using MIMIC-IV data. The prediction target was defined as vasopressor initiation and/or early death during the 24–72 h outcome window after ICU admission. This endpoint is broader than mortality prediction alone, but its interpretation should consider that most positive cases were vasopressor-related and may reflect illness severity, treatment escalation, and ICU management practices in addition to cardiovascular-specific deterioration.

The proposed model combines three early data sources: enriched structured clinical variables from the first 24 h of ICU stay, 24-h vital sign time series, and timestamp-filtered radiology reports represented using CXR-BERT-specialized embeddings. On the held-out internal test set, MultiCardioNet achieved an AUROC of 0.9014, AUPRC of 0.8481, and F1-score of 0.7723 at the validation-selected operating threshold. These results were higher than those of the evaluated unimodal baselines and the strongest early-fusion XGBoost baseline, suggesting that the three modalities provided complementary information for this prediction task.

Additional analyses support a cautious interpretation of the results. Component-level analysis showed that performance was mainly associated with vasopressor-related cases. Calibration analysis showed improved internal calibration after Platt scaling, and threshold analysis showed trade-offs between recall, precision, and alert burden. While the interpretability and subgroup analyses can provide an initial assessment of model behavior, these analyses should not be interpreted as causal explanations or a complete fairness evaluation.

Overall, the findings suggest that multimodal learning can support prediction of this composite ICU deterioration endpoint in the internal MIMIC-IV setting. However, full multimodal external validation, prospective evaluation, recalibration, and broader subgroup assessment are needed before considering clinical deployment. Future work should also investigate missing-modality learning, additional explainability methods, and integration of cardiac-specific modalities such as ECG and echocardiography when sufficient temporally aligned data are available.

## Figures and Tables

**Table 1:** Structured variable groups used in the structured baseline models.

Variable Group	Examples
Demographics	Age and sex
Vital signs	Heart rate, blood pressure, respiratory rate, oxygen saturation, temperature
Laboratory measurements	Bilirubin, creatinine, glucose, lactate, electrolytes, hemoglobin, white blood cell count, platelet count

**Table 2:** Configuration of the proposed multimodal neural fusion model.

Component	Configuration
Structured branch	rich24 clinical feature vector encoded using an MLP
Time series branch	24-h vital sign sequence encoded using a transformer encoder
Radiology branch	CXR-BERT-specialized radiology embeddings encoded using a dense neural branch
Fusion classifier	Concatenation of branch outputs followed by an MLP classifier
Output	Cardiovascular deterioration risk probability

**Table 3:** Data sources and modalities used in this study.

Data Source	Extracted Information	Model Modality
MIMIC-IV hospital module	Demographics, laboratory measurements, death time	Structured clinical data
MIMIC-IV ICU module	ICU stays, vital signs, medication events	Structured and time series data
MIMIC-IV-Note	Early radiology reports	Radiology text data

**Table 4:** Detailed content of the dataset used for model construction.

Data Type	Field or Feature Group	Description	Format
Identifiers	subject_id, hadm_id, stay_id	Patient, hospital admission, and ICU stay identifiers used for data alignment	Integer identifiers
Structured data	Demographics	Age and sex	Numeric and binary variables
Structured data	First vital signs	Early heart rate, blood pressure, respiratory rate, oxygen saturation, and temperature	Continuous variables
Structured data	First laboratory tests	Bilirubin, creatinine, glucose, lactate, potassium, sodium, hemoglobin, white blood cell count, and platelet count	Continuous variables
Structured data	rich24 descriptors	Count, first value, last value, minimum, maximum, mean, standard deviation, trend, and missingness indicators extracted from first-24-h variables	Continuous and binary variables
Time-series data	Vital-sign sequence	Heart rate, systolic blood pressure, diastolic blood pressure, mean blood pressure, respiratory rate, and oxygen saturation over 24 h	24×6 hourly sequence
Text data	Radiology reports	Early radiology report text aligned with the ICU stay	TF-IDF features or dense CXR-BERT-specialized embedding
Outcome data	Vasopressor initiation and early death	Components used to define the composite cardiovascular deterioration endpoint	Binary indicators

**Table 5:** Decomposition of the composite cardiovascular deterioration endpoint in the full cohort.

Endpoint Component	Full Cohort Count
Vasopressor-only	17,988
Death-only	669
Vasopressor + death overlap	1008
Total positive cases	19,665
Negative cases	34,886
Total ICU stays	54,551

**Table 6:** Training, validation, and test split distribution.

Split	Total Stays	Positive Cases	Negative Cases	Positive Rate
Training	34,912	12,586	22,326	36.05%
Validation	8728	3146	5582	36.05%
Test	10,911	3933	6978	36.05%

**Table 7:** Experimental models evaluated in this study.

Model Family	Input Data	Representation or Fusion Strategy
Structured LR	Structured clinical variables	Demographic, vital sign, and laboratory variables with logistic regression
Structured RF	Structured clinical variables	Demographic, vital sign, and laboratory variables with random forest
Structured XGBoost	Structured clinical variables	Demographic, vital sign, and laboratory variables with XGBoost
Time series summary XGBoost	Vital sign time series	Summary statistics from the 24-h vital sign sequence with XGBoost
Time series LSTM	Vital sign time series	Full 24-h vital sign sequence with an LSTM encoder
Time series TCN	Vital sign time series	Full 24-h vital sign sequence with a temporal convolutional encoder
Time series transformer	Vital sign time series	Full 24-h vital sign sequence with a transformer encoder
Radiology TF-IDF	Radiology reports	TF-IDF features from timestamp-filtered radiology reports
Radiology CXR-BERT	Radiology reports	CXR-BERT-specialized embeddings from timestamp-filtered radiology reports
CXR-BERT radiology embeddings + XGBoost	Radiology reports	CXR-BERT-specialized radiology embeddings with XGBoost classification
Two-modality early-fusion XGBoost	Structured + time series; structured + radiology; time series + radiology	Concatenated modality features followed by XGBoost classification
Three-modality early-fusion XGBoost	Structured + time series + radiology	Concatenated structured, time series summary, and radiology features followed by XGBoost classification
Late-fusion average	Structured + time series + radiology	Average of modality-specific prediction probabilities
Late-fusion stacking	Structured + time series + radiology	Prediction-level stacking using modality-specific model outputs
Proposed-architecture ablations	Structured + time series; structured + radiology; time series + radiology; structured + time series + radiology	Neural fusion architecture evaluated with different modality combinations
MultiCardioNet	Structured + time series + radiology	rich24 features + transformer time series encoder + CXR-BERT-specialized radiology embeddings with neural fusion

**Table 8:** Key reproducibility details for the final MultiCardioNet model.

Component	Specification
Primary dataset	MIMIC-IV version 3.1
Clinical notes	MIMIC-IV-Note version 2.2 radiology reports
Observation window	0–24 h after ICU admission
Prediction time	24 h after ICU admission
Outcome window	24–72 h after ICU admission
Endpoint	Vasopressor initiation and/or death during the outcome window
Early-event exclusion	Vasopressor initiation or death during 0–24 h
Vasopressor definitions	Norepinephrine/Levophed, epinephrine/adrenaline, phenylephrine/Neo-Synephrine, vasopressin, dopamine, dobutamine
Early death definition	Recorded death during the 24–72 h outcome window
Radiology time filter	Storage/finalization time used for retained reports; chart time considered only as fallback during preprocessing
Positive-case radiology rule	Reports retained before the first recorded outcome event or before the 24-h observation cutoff, whichever occurred first
Negative-case radiology rule	Reports retained only within the first 24 h after ICU admission
Radiology encoder	microsoft/BiomedVLP-CXR-BERT-specialized
Radiology embedding	CLS token, maximum length 512 tokens, frozen encoder, TruncatedSVD to 256 dimensions
Structured input	256 structured clinical features
Time series input	24 × 6 hourly vital sign tensor
Optimizer	AdamW
Learning rate	$1\times 10^{-4}$
Weight decay	$1\times 10^{-4}$
Batch size	192
Loss function	Binary cross-entropy with logits loss using positive-class weighting
Dropout	0.30
Early stopping	Validation AUPRC, patience 15 epochs
Maximum epochs	100
Split seed	42
Model seeds	42, 7, 123
Primary reported model seed	7
Software	Python 3.12.13, PyTorch 2.6.0+cu124, Hugging Face Transformers 5.13.1, scikit-learn 1.8.0, XGBoost 3.2.0, NumPy 2.4.3, pandas 3.0.2

**Table 9:** Comparison of representative baseline, fusion, and MultiCardioNet models on the held-out test set.

Model	Type	Threshold	AUROC	AUPRC	Accuracy	Precision	Recall	F1-Score
Structured LR	Structured	0.475	0.7603	0.6258	0.6917	0.5546	0.7353	0.6323
Structured RF	Structured	0.410	0.8220	0.7170	0.7352	0.6023	0.7813	0.6802
Structured XGBoost	Structured	0.460	0.8329	0.7193	0.7426	0.6096	0.7953	0.6902
Time-series summary XGBoost	Time-series	0.510	0.8334	0.7255	0.7516	0.6228	0.7885	0.6959
Time-series LSTM	Time-series	0.370	0.8227	0.7156	0.7152	0.5715	0.8398	0.6801
Time-series TCN	Time-series	0.215	0.7773	0.6599	0.6713	0.5290	0.8042	0.6382
Time-series Transformer	Time-series	0.370	0.8263	0.7228	0.7242	0.5819	0.8347	0.6857
Radiology TF-IDF	Radiology text	0.515	0.8038	0.7115	0.7758	0.7086	0.6423	0.6738
Radiology CXR-BERT	Radiology text	0.460	0.7852	0.7017	0.7444	0.6409	0.6616	0.6511
CXR-BERT radiology embeddings + XGBoost	Radiology text	0.440	0.6897	0.4848	0.6122	0.4792	0.8739	0.6190
Structured + time-series XGBoost	Multimodal (S + T), early fusion	0.480	0.8661	0.7700	0.7780	0.6537	0.8169	0.7263
Structured + radiology XGBoost	Multimodal (S + R), early fusion	0.490	0.8662	0.7699	0.7939	0.6828	0.7996	0.7366
Time-series + radiology XGBoost	Multimodal (T + R), early fusion	0.510	0.8636	0.7730	0.7805	0.6619	0.7994	0.7242
Structured + time-series + radiology XGBoost	Multimodal (S+T+R), early fusion	0.560	0.8905	0.8070	0.8210	**0.7354**	0.7864	0.7600
Late-fusion average (S + T + R)	Multimodal (S + T + R), late fusion	0.520	0.8787	0.7879	0.8049	0.7041	0.7913	0.7451
Late-fusion stacking (S + T + R)	Multimodal (S + T + R), late fusion	0.510	0.8786	0.7877	0.8019	0.6927	0.8093	0.7465
**MultiCardioNet**	Multimodal (S + T + R), neural fusion	0.590	**0.9014**	**0.8481**	**0.8256**	0.7294	**0.8205**	**0.7723**

S = structured clinical features; T = time series features; R = radiology text features. Late-fusion average and late-fusion stacking are multimodal fusion baselines that combine modality-specific predictions, not variants of the MultiCardioNet architecture. Bold text identifies the best-performing values and the final MultiCardioNet model.

**Table 10:** ICU stay-level bootstrap 95% confidence intervals and paired comparison with the strongest baseline structured + time series + radiology XGBoost early fusion model on the held-out test set.

Metric	MultiCardioNet	S + T + R XGBoost Baseline	Difference	*p*-Value
AUROC	0.9014 (0.8955–0.9073)	0.8905 (0.8844–0.8966)	0.0109 (0.0068–0.0152)	<0.001
AUPRC	0.8481 (0.8373–0.8586)	0.8070 (0.7929–0.8198)	0.0411 (0.0314–0.0519)	<0.001
Accuracy	0.8256 (0.8186–0.8326)	0.8205 (0.8132–0.8278)	0.0050 (−0.0010–0.0113)	0.100
Precision	0.7363 (0.7229–0.7491)	0.7324 (0.7194–0.7458)	0.0039 (−0.0050–0.0135)	0.415
Recall	0.8042 (0.7916–0.8162)	0.7913 (0.7786–0.8034)	0.0130 (0.0013–0.0240)	0.025
Specificity	0.8376 (0.8288–0.8460)	0.8371 (0.8284–0.8454)	0.0006 (−0.0067–0.0084)	0.891
NPV	0.8836 (0.8759–0.8912)	0.8768 (0.8687–0.8847)	0.0068 (0.0008–0.0127)	0.025
F1-score	0.7687 (0.7586–0.7782)	0.7607 (0.7503–0.7711)	0.0081 (0.0000–0.0166)	0.050

**Table 11:** MultiCardioNet stability across random seeds on the held-out test set.

Seed	Threshold	AUROC	AUPRC	Accuracy	Recall	F1-Score
42	0.63	0.8988	0.8455	0.8313	0.7803	0.7693
7	0.59	0.9014	0.8481	0.8256	0.8205	0.7723
123	0.58	0.9005	0.8490	0.8275	0.8075	0.7714
Mean ± SD	–	0.9003 ± 0.0013	0.8475 ± 0.0018	0.8281 ± 0.0029	0.8028 ± 0.0205	0.7710 ± 0.0016

**Table 12:** Calibration metrics for uncalibrated and Platt-calibrated MultiCardioNet probabilities on the held-out test set.

Probability Output	Brier Score	ECE	MCE	Calibration Intercept	Calibration Slope	Mean Predicted Risk
Uncalibrated	0.1319	0.0885	0.2227	−0.6902	0.8193	0.4434
Platt-calibrated	0.1185	0.0143	0.0276	−0.0052	1.0075	0.3614

**Table 13:** Observed versus predicted risk by decile for Platt-calibrated MultiCardioNet probabilities on the held-out test set.

Risk Decile	Test Cases	Positive Cases	Mean Predicted Risk	Observed Event Rate
1	1092	26	0.0082	0.0238
2	1091	41	0.0267	0.0376
3	1091	52	0.0522	0.0477
4	1091	99	0.0986	0.0907
5	1091	177	0.1802	0.1622
6	1091	342	0.3247	0.3135
7	1091	526	0.5098	0.4821
8	1091	724	0.6703	0.6636
9	1091	903	0.8062	0.8277
10	1091	1043	0.9374	0.9560

**Table 14:** Multimodal ablation analysis within the proposed neural architecture on the held-out test set.

Proposed Architecture Mode	Threshold	AUROC	AUPRC	Accuracy	F1-Score	Recall	Precision
Structured + time-series	0.54	0.8840	0.8074	0.8010	0.7485	0.8215	0.6874
Structured + radiology text	0.48	0.8923	0.8398	0.8194	0.7648	0.8146	0.7206
Time-series + radiology text	0.52	0.8750	0.7979	0.7850	0.7356	0.8299	0.6606
**MultiCardioNet**	0.59	0.9014	0.8481	0.8256	0.7723	0.8205	0.7294

Bold text identifies the full MultiCardioNet configuration.

**Table 15:** MultiCardioNet performance on the held-out test set stratified by availability of eligible radiology text.

Subgroup	Test Cases	AUROC	AUPRC	Accuracy	Recall	F1-Score
With eligible radiology text	4236	0.8277	0.5453	0.8407	0.5364	0.5216
Without eligible radiology text	6675	0.9021	0.8944	0.8156	0.8750	0.8219

**Table 16:** Predictive value of eligible radiology text availability alone, evaluated on the held-out test set.

Model	AUROC	AUPRC	Accuracy	Precision	Recall	F1-Score
Radiology-availability indicator only	0.6808	0.4788	0.6175	0.4825	0.8467	0.6147

**Table 17:** Component-level analysis of the composite cardiovascular deterioration endpoint on the held-out test set.

Endpoint Comparison	Positive Cases	Threshold	AUROC	AUPRC	Recall	F1-Score
Composite endpoint	3933	0.41	0.9014	0.8481	0.8159	0.7710
Vasopressor-only vs. negatives	3576	0.41	0.9039	0.8406	0.8224	0.7640
Death-only vs. negatives	152	0.72	0.7773	0.1105	0.2237	0.1481
Vasopressor + death vs. negatives	205	0.81	0.9491	0.4847	0.4829	0.4737

**Table 18:** Validation-selected operating points for MultiCardioNet on the held-out test set.

Operating Point	Threshold	Accuracy	PPV	Recall	Specificity	NPV	F1-Score	Alert Burden
Balanced validation-selected threshold	0.41	0.8253	0.7308	0.8159	0.8306	0.8890	0.7710	40.24%
Lower-alert operating point	0.60	0.8286	0.8163	0.6768	0.9142	0.8339	0.7401	29.89%

**Table 19:** Permutation-based modality importance for MultiCardioNet on the held-out test set.

Input Modality	Repeats	AUROC Decrease	AUPRC Decrease
Structured features	5	0.1915	0.3120
Radiology text	5	0.0641	0.0929
Time-series features	5	0.0491	0.0680

**Table 20:** Top structured features by permutation-based AUPRC decrease on the held-out test set.

Structured Feature	AUROC Decrease	AUPRC Decrease
Minimum systolic blood pressure	0.0093	0.0129
Glucose standard-deviation missingness indicator	0.0023	0.0061
Temperature standard-deviation missingness indicator	0.0017	0.0049
Minimum heart rate	0.0030	0.0039
Lactate measurement count	0.0023	0.0038
Glucose measurement count	0.0011	0.0030
Sodium standard-deviation missingness indicator	0.0010	0.0028
Lactate standard-deviation missingness indicator	0.0015	0.0020

**Table 21:** Permutation-based time series importance on the held-out test set.

Time Series Input	Repeats	AUROC Decrease	AUPRC Decrease
Diastolic BP	5	0.0309	0.0427
Systolic BP	5	0.0197	0.0257
Mean BP	5	0.0107	0.0158
Heart rate	5	0.0052	0.0105
SpO_2_	5	0.0045	0.0070
Respiratory rate	5	0.0032	0.0038
Hours 0–4	5	0.0066	0.0104
Hours 16–20	5	0.0072	0.0100
Hours 20–24	5	0.0074	0.0086
Hours 4–8	5	0.0044	0.0063
Hours 8–12	5	0.0033	0.0046
Hours 12–16	5	0.0036	0.0044

**Table 22:** Representative radiology terms associated with higher and lower surrogate-predicted MultiCardioNet risk.

Direction	Representative Radiology Terms
Higher surrogate-predicted risk	shock, arrest, sepsis, cardiac arrest, Swan-Ganz, hypotension, catheter terminates, coronary artery, IABP, severe
Lower surrogate-predicted risk	clear, normal, improved, removal, removed, low, right lung, left basilar, tracheostomy, atelectatic changes

**Table 23:** Subgroup performance of MultiCardioNet on the held-out test set using the validation-selected threshold.

Subgroup	Test Cases	Positive Rate	AUROC	AUPRC	Accuracy	PPV	Recall	F1-Score
Age <65	5205	31.47%	0.9011	0.8221	0.8300	0.6995	0.8059	0.7489
Age 65–79	3690	42.49%	0.8999	0.8756	0.8228	0.7685	0.8342	0.8000
Age ≥80	2016	36.06%	0.8988	0.8444	0.8180	0.7244	0.7992	0.7600
Female	4661	32.20%	0.8969	0.8220	0.8245	0.7036	0.7861	0.7426
Male	6250	38.91%	0.9035	0.8635	0.8259	0.7476	0.8343	0.7886
With eligible radiology text	4236	16.19%	0.8277	0.5453	0.8407	0.5076	0.5364	0.5216
Without eligible radiology text	6675	48.64%	0.9021	0.8944	0.8156	0.7750	0.8750	0.8219

**Table 24:** Subgroup performance by ICU care unit and simplified race group on the held-out test set.

Subgroup	Test Cases	Positive Rate	AUROC	AUPRC	Recall	F1-Score
**First ICU care unit**						
CVICU	2259	68.39%	0.8969	0.9499	0.9482	0.8759
MICU	2000	31.50%	0.8776	0.7728	0.7746	0.7230
MICU/SICU	1572	28.50%	0.8813	0.7569	0.7478	0.7060
SICU	1492	24.66%	0.8734	0.7379	0.7092	0.6905
CCU	1204	34.80%	0.8592	0.7426	0.7208	0.7073
TSICU	1204	32.23%	0.8788	0.7648	0.7680	0.7233
Neuro Intermediate	754	3.85%	0.8695	0.3690	0.4828	0.3889
Neuro SICU	216	29.63%	0.7981	0.6269	0.5781	0.5736
Neuro Stepdown	167	8.38%	0.9034	0.7899	0.7143	0.7143
**Simplified race group**						
White	7181	37.04%	0.9070	0.8608	0.8267	0.7828
Unknown/Not reported	1580	41.14%	0.8756	0.8335	0.8231	0.7594
Black	978	25.15%	0.8971	0.7756	0.7805	0.7245
Other	483	35.61%	0.8773	0.8208	0.8081	0.7658
Hispanic/Latino	363	30.85%	0.9280	0.8615	0.8393	0.7833
Asian	326	28.53%	0.8705	0.7909	0.7312	0.6904

Bold text identifies subgroup category headers.

## Data Availability

The data used in this study are openly available through the MIMIC-IV and MIMIC-IV-Note databases on PhysioNet: MIMIC-IV, https://doi.org/10.13026/kpb9-mt58; MIMIC-IV-Note, https://doi.org/10.13026/1n74-ne17.

## Abbreviations

The following abbreviations are used in this manuscript:

AUPRC	Area Under the Precision–Recall Curve
AUROC	Area Under the Receiver Operating Characteristic Curve
BCE	Binary Cross-Entropy
DBP	Diastolic Blood Pressure
ECG	Electrocardiography
EHR	Electronic Health Record
FN	False Negative
FP	False Positive
HR	Heart Rate
ICU	Intensive Care Unit
LR	Logistic Regression
MAP	Mean Arterial Pressure
MIMIC-IV	Medical Information Mart for Intensive Care IV
MLP	Multilayer Perceptron
PR	Precision–Recall
ROC	Receiver Operating Characteristic
RR	Respiratory Rate
SBP	Systolic Blood Pressure
SpO_2_	Peripheral Oxygen Saturation
TF-IDF	Term Frequency–Inverse Document Frequency
TN	True Negative
TP	True Positive
